# The moderating role of anger rumination and self‐control in the day‐to‐day associations between irritability and sleep in male adolescents: An exploratory experience sampling method study

**DOI:** 10.1002/jcv2.70183

**Published:** 2026-10-29

**Authors:** Julia Giovannini, Setareh Ranjbar, Jennifer Glaus, Ines Mürner‐Lavanchy, Lauriane Constanty, Kerstin Jessica Plessen, Sébastien Urben

**Affiliations:** ^1^ Division of Child and Adolescent Psychiatry Department of Psychiatry Lausanne University Hospital and University of Lausanne Lausanne Switzerland; ^2^ Faculty of Biology and Medicine University of Lausanne Lausanne Switzerland; ^3^ Center of Psychiatric Epidemiology and Psychopathology Department of Psychiatry Lausanne University Hospital and University of Lausanne Lausanne Switzerland; ^4^ Division of Youth Mental Health Faculty of Psychology University of Basel Basel Switzerland

**Keywords:** adolescents, ecological momentary assessment, emotion regulation, irritability, self‐regulation, sleep disturbances

## Abstract

**Background:**

Irritability is highly prevalent in adolescents and may be exacerbated by sleep disturbances, with sleep patterns undergoing significant changes during this developmental period. Reciprocally, poor sleep may generate an increase in anger dysregulation. This exploratory study investigates the day‐to‐day associations between sleep and irritability, as well as the moderating effects of maladaptive (i.e., anger rumination) and adaptive (i.e., self‐control) emotion regulation (ER) strategies.

**Methods:**

Fifty‐six male adolescents aged 12–18, with diverse sleep and emotional problems, participated in a 9‐day experience sampling method study. Participants also completed self‐report questionnaires assessing irritability, sleep quantity and quality, and maladaptive and adaptive ER strategies. Linear mixed effect models with lagged covariates were used to test associations and moderating effects.

**Results:**

At the within‐person level, sleep quality was associated with next‐day irritability (*β*std = −0.92, 95% CI [−1.31, −0.53). Higher daily anger rumination (*β*std = 0.25, 95% CI [0.17, 0.34]), and daily self‐control (*β*std = −0.18, 95% CI [−0.27, −0.10]) were directly associated with higher next‐day irritability. At the between‐person level, lower self‐control was also associated with higher next‐day irritability (*β*std = −0.52, 95% CI [−0.77, −0.27]). Interactions between daily sleep quality and between‐person anger rumination (${\beta \text{std}}_{\text{interaction}}$ = 0.18, 95% [0.07, 0.30]), and self‐control (${\beta \text{std}}_{\text{interaction}}$ = 0.75, 95% CI [0.38, 1.12]) were related with irritability. Reciprocally, lower irritability from the day was associated with better sleep quality the following night (*β*std = −0.11, 95% CI [−0.20, −0.01]).

**Conclusion:**

These preliminary findings suggest that sleep may be related to next‐day irritability in adolescent boys and may vary as a function of anger rumination and self‐control. Additionally, irritability also showed a potential association with subsequent sleep. However, findings should be interpreted cautiously given the small, all‐male adolescent sample. Future studies including larger and more diverse adolescent samples are needed to assess their generalizability and robustness.

## Key Points

What's known?

- Irritability in adolescents is a major public health concern and is closely associated with sleep disturbances, yet the day‐to‐day relationship between these two constructs remains poorly understood.

What's new?

- Few studies have examined how anger rumination (a maladaptive emotion regulation [ER] strategy) and self‐control (an adaptive ER strategy) contribute to these associations, and, to our knowledge, none have explored all four variables together.
- Our preliminary findings suggest that the association between sleep and irritability may exist and may differ according to levels anger rumination and self‐control at the between‐person level, while the reverse association was modest and warrants replication.

What's relevant?

- This exploratory study highlights the potential relevance of considering both sleep habits and ER processes in male adolescence and support further investigation of interventions targeting sleep hygiene, ER skills, and irritability.

## INTRODUCTION

Irritability is one of the most common causes of referral to mental health services (Evans et al., 2023) and one of the most widespread symptoms across mental health disorders described in the Diagnostic and Statistical Manual of Mental Disorders, 5th edition (DSM‐5) (American Psychiatric Association, 2013; Klein et al., 2021; Toohey & DiGiuseppe, 2017). It may be defined as a persistent negative mood and characterized by excessive reactivity to negative stimuli and a lowered threshold to frustration tolerance (Leibenluft & Stoddard, 2013; Vidal‐Ribas et al., 2016). Additionally, it may occur without clearly identified triggers such as fatigue, stress or physical pain (Grondal et al., 2023). A distinction with anger can be made, as anger refers to an emotion or a feeling that is often tied to specific, identifiable events (Grondal et al., 2023). To sum up, irritability may be understood as a chronic proneness to anger and as a dispositional tendency, whereas anger is the acute, momentary episode. Indeed, anger outbursts or spikes of state anger may occur on top of an irritable mood (Jha et al., 2021). In an intensive longitudinal study, anger has been shown to emerge as a central momentary symptom within irritability networks (Tseng et al., 2023).

Irritability is highly prevalent in children and adolescents, where pubertal stage as well as environmental, social, biological, and psychological factors play a crucial role (Bell et al., 2021; Kessel et al., 2021; Klein et al., 2021). It appears early in development, with angry facial expressions observable as early as 2 months of age (Lewis, 1990). A marked increase is typically seen during toddlerhood, a period that may reveal distinct risk trajectories for later psychopathology (Zhang et al., 2024). These trajectories may intensify or persist during adolescence, as this developmental stage is characterized by increased vulnerability to internalizing and externalizing disorders such as depressive disorder, anxiety disorders, oppositional defiant disorder, and attention‐deficit/hyperactivity disorder (Chad‐Friedman et al., 2021; Patel et al., 2021; Yu et al., 2023; Zhang et al., 2024).

Moreover, irritability has also been associated with sleep disturbances during adolescence, as this period of life represents a “perfect storm”, arising from the convergence of multiple developmental, environmental, and psychosocial factors (Carskadon, 2011; Crowley et al., 2018). These include shifts in sleep circadian rhythm, poor sleep hygiene, and academic pressure, which may impair brain development, physical health, and quality of life (Anastasiades et al., 2022; Chattu et al., 2018; Delahoyde et al., 2024; Kansagra, 2020). For instance, while adolescents require 8–10 h of sleep per night (Medicine, 2016), most sleep less than 8 h on school nights. This discrepancy, along with sleep duration differences between weekdays and weekends (i.e., weekend oversleep, early rise time and late bedtime on weekdays) is associated with negative mental health outcomes (Kim et al., 2022).

More specifically, insufficient sleep quality, including dissatisfaction, disrupted continuity (i.e., inability to maintain sleep without interruptions), and prolonged sleep latency, can lead to negative outcomes such as depressive symptoms, disrupted emotional experiences, fatigue, reduced attention, impaired cognitive functioning, anxiety, and enhanced irritability (Chattu et al., 2018; Nelson et al., 2022; Parsons et al., 2022). Conversely, irritability may also cause sleep disturbances, with sleep deprivation and insomnia being associated with mood and anxiety disorders (Uccella et al., 2023). Nevertheless, the temporal relationship between sleep and the development of irritability has been insufficiently explored, particularly in real‐life settings, thereby limiting a comprehensive understanding of the phenomenon (Meltzer, 2017).

Irritability is also closely related to emotion regulation (ER), which can be described as “the extrinsic and intrinsic processes responsible for monitoring, evaluating, and modifying emotional reactions, especially their intensive and temporal features, to accomplish one's goals” (Thompson, 1994). In particular, maladaptive ER strategies, such as self‐blame, rumination, catastrophizing, and blaming others, were reported to be associated with psychopathologies (Aldao & Nolen‐Hoeksema, 2012; Kraaij & Garnefski, 2012), and more specifically with irritability (Derella et al., 2019). One particularly interesting maladaptive ER strategy, in the context of studying irritability, is anger rumination (Garnefski et al., 2001). It refers to the constant thinking about a past anger‐inducing experience that is meaningful to an individual (Denson, 2013). A prospective study of 165 youths aged 12–14 demonstrated that anger rumination was significantly associated with irritability 6 months later, controlling for baseline irritability and depressive rumination (Leigh et al., 2020). The emotional cascade model has been proposed to explain the temporal dynamics of anger rumination, theorizing that negative emotion increases rumination, which in turn intensifies negative emotion, resulting in an exponentially aggravating negative loop (Selby et al., 2016). One crucial mechanism allowing the regulation of negative emotions is self‐control, which can be defined as “the ability or capacity to overcome or resist impulses and temptations and modify behavior through active and conscious means” (Hagger, 2010). It is considered an executive function, involving inhibitory control, cognitive flexibility, and working memory, all of which contribute to the capacity to regulate emotional responses in the achievement of long‐term goals (Diamond, 2013). Therefore, studies have demonstrated that self‐control might be particularly important in breaking the cycle of anger rumination (Denson, 2013; Denson et al., 2012), although anger rumination may itself deplete self‐control resources (Lievaart et al., 2017). In addition, low self‐control has been theorized to be associated with diminished tolerance to frustration, meaning that minor annoyances or events are more likely to trigger disproportionate emotional reactions (Lazarus & Costa, 2020), which may result in heightened levels of irritability (Wiese et al., 2020). Conceptually, sleep and irritability may be understood as mutually influencing processes, as reduced sleep may be associated with heightened emotional reactivity, whereas elevated irritability, in turn, is linked to poorer sleep quality (Meigs et al., 2024; Whiting et al., 2023). Within these reciprocal associations, anger rumination may function as a vulnerability factor by reinforcing negative affective states, thereby strengthening the link between sleep disturbances and irritability (Boon et al., 2023; Li et al., 2019). Similarly, the tendency to ruminate on anger‐eliciting events may contribute to prolonged arousal before bedtime, which may be associated with longer sleep onset latency and lower sleep quality (Shao et al., 2025; Watkins & Roberts, 2020). In contrast, self‐control might operate as a protective factor that attenuates these associations. Indeed, higher levels of self‐control have been associated with better sleep quality and fewer negative emotional and aggressive outcomes, suggesting that this construct could potentially weaken the sleep‐irritability association (Liu et al., 2020; Wang et al., 2024; Ye et al., 2024). Building on prior research that conceptualizes rumination and self‐control as stable traits, we treated these two constructs as between‐person moderators (Plessen et al., 2023; Takebe et al., 2016; Turri et al., 2024).

In terms of sex differences during adolescence, several studies suggest that irritability, sleep patterns, and ER processes may operate differently in boys and girls during this period of life. Girls generally show higher internalizing symptoms, whereas boys more commonly exhibit externalizing manifestations of irritability (Humphreys et al., 2019; Vidal‐Ribas et al., 2025). Regarding sleep, girls report shorter sleep duration and poorer sleep quality, while boys show different developmental trajectories characterized by later bedtimes and circadian shifts (Duston et al., 2025). Concerning ER, some studies indicate that girls engage more frequently in anger rumination, whereas boys rely more often on suppression strategies (Alloy et al., 2016; Levkovich et al., 2025; Sanchis‐Sanchis et al., 2020). More broadly, girls may depend more on cognitive strategies, both adaptive or maladaptive, while boys more frequently use externalizing forms of regulation as well as acceptance in daily life (te Brinke et al., 2020). Regarding self‐control, research suggested that girls typically report having higher levels of self‐control skills than boys (Tetering et al., 2020). Together, these findings support the examination of these constructs within a homogeneous sample of adolescent boys in the current study.

Despite extensive research, the different associations between sleep and irritability, as well as the moderating role of ER and self‐control in these relationships remains unclear (Baum et al., 2014; Liu et al., 2020; Whiting et al., 2023). Some studies observed associations between poor sleep and heightened irritability or negative affect, yet many relied on cross‐sectional designs, raising recall bias concerns (Baum et al., 2014; Palmer & Alfano, 2017; Short & Louca, 2015). Additionally, much of the existing research focuses on the unidirectional effect of a poor night of sleep on emotions experienced the following day, without examining the inverse association (Parsons et al., 2022). Finally, these four constructs have often been studied in isolation, rather than through an integrative approach, which would provide a more comprehensive picture of their interrelationships.

To address these limitations, an ecological approach such as the experience sampling method (ESM) may be particularly useful. ESM involves repeated self‐report assessments of emotions, behaviors, thoughts, and experiences throughout the day using an electronic device, typically a smartphone (Myin‐Germeys & Kuppens, 2022). This methodology also offers the examination of within‐ and between‐person variability by capturing emotion dynamics in real‐life settings (Silver et al., 2025). Indeed, examining the temporal fluctuations of emotions over an extended period may highlight emotional stability while also allowing for the exploration of temporal dynamics (i.e., lagged effects) (Kuppens et al., 2010). Although irritability is often conceptualized as a relatively stable dispositional tendency, the present study mostly focuses on within‐person fluctuations in its daily experience, which may vary in response to contextual and regulatory processes.

### The current study

The present study takes an exploratory approach to examine the day‐to‐day relationships between sleep and irritability among adolescent boys using ESM. We further explored the moderating role of anger rumination (a maladaptive ER strategy) and self‐control (an adaptive ER strategy) on these associations.

## MATERIALS AND METHODS

### Participants

A total of 62 male participants with varying degrees of emotional and sleep problems were enrolled in the study. Six participants were excluded due to a large amount of missing data (>50%), leaving a final sample of 56 individuals aged 12 and 18 years (*M* = 15.25; SD = 1.45) (Table 1). A dimensional approach was chosen, as it allows for a more nuanced understanding of psychological phenomena by positioning adolescents along a continuum of emotional and sleep‐related difficulties (Fernandez, 2019). Only male participants were included, as the global study focused on externalizing symptoms, such as aggression, impulsivity, and emotional dysregulation which are more commonly associated with males than females (Martel, 2013; Van Veen et al., 2021). Moreover, this sampling approach allows for a more controlled examination of underlying mechanisms of irritability without the confounding influence of sex and gender‐specific variations (Humphreys et al., 2019). We recruited participants through advertisements on websites dedicated to youth, as well as in schools and other institutions, such as residential care institutions. To be included in the study, participants had to have access to a smartphone. They also needed a sufficient level of French and adequate intellectual abilities. In the original study, exclusion criteria were diverse (Plessen et al., 2023). Young people who used psychotropic medication and had diagnoses of schizophrenia, psychosis, and/or autism spectrum disorder were excluded from the pool of participants, since these factors can influence affective states (Paulus et al., 2021; Solmi et al., 2020).

**TABLE 1:** Socio‐demographic characteristics (*n* = 56) (upper‐part) and descriptive statistics of baseline variables obtained through self‐report questionnaires (between‐person) and variables obtained through ESM (within‐person).

Variable	Level	*M* (SD)/%(*n*)
Age (years)		15.25 (1.45)
Socio‐economic level	Low	14.3 (8)
	Medium	37.5 (21)
	High	41.07 (23)
Nationality	Swiss	85.71 (48)
First language	French	89.28 (50)
Ongoing education		96.43 (54)
Education	Primary school	35.71 (20)
	Secondary school	58.93 (33)
	Other	5.36 (3)
Living	At home	80.36 (45)
	In institution	16.07 (9)
	Other	3.57 (2)
Between‐person (trait)		
Trait maladaptive ER		32.54 (8.83)
Trait sleep difficutlties		0.90 (0.47)
Trait sleep quantity		7.61 (0.96)
Trait irritability		0.83 (0.54)
Trait self‐control		40.96 (7.16)
Within‐person (state)		
Daily irritability		11.1 (14.83)
Sleep quality		4.30 (1.59)
Daily anger rumination		12.06 (22.93)
Daily self‐control		74.31 (18.36)

*Note*: Within‐person variables are averaged daily and then averaged over a period of 9 days.

Abbreviations: ER, emotion regulation; ESM, experience sampling method.

### Procedure

After we received authorization from the ethics committee of the Canton of Vaud (#2019‐02318), participants were provided with oral and written information about the study. Both the adolescents and their legal representatives gave their consent to participate through a written consent form. The procedure followed a two‐stage process, consisting first of a baseline assessment to examine between‐person (trait level) variables and second of an ambulatory assessment to capture within‐person (state level) variables.

The ESM protocol involved short questionnaires (approximately 4 min long) prompted four times a day (7 a.m., 12 p.m., 4 p.m., and 8 p.m.) over 9 days. Weekends were excluded to minimize the risk of non‐random missingness and reduced adherence, which may arise due to participants ignoring prompts during leisure time or following irregular weekend schedules (Tate et al., 2024; Wrzus & Neubauer, 2023). SMS messages sent to participants' smartphones contained the link to the Redcap^®^ survey. At the end of the protocol, participants received 100 CHF as compensation for their participation.

### Measures

#### Baseline measures (trait level)

##### Sociodemographic

Sociodemographic measures included age, nationality, language, education level, living status, and socioeconomic status (SES) based on the parents' respective professions. Highest degree of education was obtained during the administration of the Kiddie Schedule for Affective Disorders and Schizophrenia (K‐SADS‐PL) (Kaufman et al., 2018). SES scores were calculated as the mean of both parents' highest degree of education and classified into three groups: “high”, “middle”, and “low”.

##### Irritability

Trait irritability was measured using the validated French version of the Youth Self‐Report (YSR), derived from the Child Behavior Checklist (Vermeersch & Fombonne, 1997; Vreugdenhil et al., 2006). This instrument consists of 112 items assessing internalizing and externalizing symptoms of psychopathology in adolescents. Different dimensions are presented, such as attention problems, social problems, thought problems, somatic complaints, mood and anxiety, delinquent and aggressive behaviors, and withdrawal. As we focused on irritability, we averaged values of three items to create a single score: “I am stubborn,” “My moods or feelings change suddenly” and “I have a hot temper,” in line with previous research (Dougherty et al., 2021; Evans et al., 2020). These items were rated on a 3‐point Likert scale, where 0 = “Not True,” 1 = “Somewhat or Sometimes True,” and 2 = “Very True or Often True” (*α* = .57).

##### Sleep

One self‐report item assessed sleep quantity (i.e., sleep duration), as reported in the sociodemographic questionnaire. Additionally, three items from the YSR questionnaire were aggregated to create a single overall trait sleep difficulties score, consistent with the operationalization used in prior research (Gregory et al., 2011): “I sleep less than most kids,” “I sleep more than most kids during the day and/or night,” and “I have trouble sleeping.” Scores were rated on the same 3‐point Likert scale as the irritability measure (0 = “Not True,” 1 = “Somewhat or Sometimes True,” 2 = “Very True or Often True”) (*α* = .39).

##### Emotion regulation

Emotion regulation strategies were measured using the validated French version of the Cognitive Emotion Regulation Questionnaire (d’Acremont & Van der Linden, 2007), a 36‐item self‐report questionnaire designed to evaluate nine adaptive and maladaptive ER strategies (*α* = .68–.78) (d’Acremont & Van der Linden, 2007). The maladaptive ER strategies included self‐blame, other‐blame, rumination, and catastrophizing. The four scores were summed to obtain a total maladaptive ER score. Responses were rated on a 5‐point Likert scale ranging from 1 = “Almost never” to 5 = “Almost always”. In this study, we focused only on the maladaptive ER scale.

##### Self‐control

The French version of the Brief Self‐Control Scale (BSCS) (Brevers et al., 2017) was used to assess self‐control. This scale includes 13 items measuring impulse control, ER, personal discipline, planning, and perseverance to achieve long‐term goals. Participants rated each item on a 5‐point Likert scale ranging from 1 = “Not at all” to 5 = “Very much” (*α* = .69–.85 [Ferrari et al., 2009; Tangney et al., 2004]). A total score was calculated by summing the responses to all items.

#### ESM measures (state level)

##### Daily irritability

Daily irritability was operationalized with an item about anger (“At this moment, I feel angry/upset”; Houben et al., 2017), representing the consciously processed emotion characterizing irritability. Indeed, a study exploring the associations between irritability, anger, and aggression has demonstrated that irritability may overlap with anger, suggesting that anger could be a primary emotional component of irritability (Zik et al., 2022). Nevertheless, we acknowledge that this operationalization may capture aggregated state anger rather than a fully distinct measure of irritability. The item was rated on a visual analog scale ranging from 0 = “no, not all” to 100 = “yes, absolutely”. The scores obtained at the four measurement points during the day were then averaged to obtain a daily irritability score.

##### Sleep quality

As part of the ESM, participants answered a daily question about their sleep quality during the morning assessment: “How rested did you feel when you woke up this morning? (Vroegindeweij et al., 2022)”. Responses were collected on a 7‐point Likert scale, where 1 = “not rested at all”, 4 = “moderately rested”, and 7 = “completely rested”. As this item was presented once daily in the morning, this score reflected the daily level for each participant.

##### Daily anger rumination

Because only maladaptive ER strategies were assessed in the ESM protocol, the present analyses focused exclusively on anger rumination. Daily anger rumination was evaluated by averaging responses to two items from the Anger Rumination Scale (Sukhodolsky et al., 2001). This questionnaire assesses maladaptive, repetitive cognitions related to anger (i.e., angry afterthoughts, thoughts of revenge, angry memories, and understanding of causes): (“Since the last questionnaire, I’ve ruminated/thought about my past angry experiences”, “Since the last questionnaire, I’ve analyzed the events that made me angry”). Importantly, these items assessed the presence and intensity of ruminative thoughts about anger, irrespective of whether a specific anger‐eliciting event had occurred during the assessment interval. Responses were recorded on a visual analog scale ranging from 0 = “no, not all” to 100 = “yes, absolutely” (*α* = .79). Scores obtained throughout the day were averaged to generate a daily value of anger rumination.

##### Daily self‐control

Daily self‐control was assessed using four items averaged to compute a composite score (“Since the last questionnaire, I was able to follow my plans and objectives”, “Since the last questionnaire, I was able to concentrate on the tasks in hand without being distracted”, “Since the last questionnaire, I lost control of myself”, “Since the last questionnaire, I easily resisted the temptation/my desires of the moment”). Items were adapted from the effortful subscale of the revised early adolescent temperament questionnaire (Ellis & Rothbart, 2001), the Barratt Impulsiveness Scale (Rouselle & Vigneau, 2016), and the BSCS (Brevers et al., 2017). They measured, respectively, the ability to stay focused on tasks over a period; attention; and loss of control over emotions, feelings, and behaviors. Participants responded using a visual analog scale ranging from 0 to 100 (*α* = .66). The four values collected throughout the day were averaged to obtain a daily self‐control score.

### Data analyses

Descriptive statistics on the continuous variables of interest obtained through ambulatory assessment (i.e., within‐person [wp] level), namely daily irritability, sleep quality, daily anger rumination, and daily self‐control, were also provided in the same setting. A correlation plot of the baseline variables using Spearman's correlation was provided, as some variables did not follow a Gaussian distribution.

Daily irritability, anger rumination, and self‐control were calculated by averaging the four measures collected throughout the day for each participant. This aggregation was chosen to capture day‐level psychological states rather than momentary reactivity and to improve the signal‐to‐noise ratio of the measures. All ESM predictors were decomposed into between‐person and within‐person components. Between‐person variables were computed as each participant's mean across observations, reflecting stable individual differences across the study period. Within‐person variables were person‐mean centered to reflect momentary deviations from an individual's own average. Lagged predictors were computed using these person‐mean‐centered within‐person variables, such that lagged effects represent prior within‐person deviations. Specifically, irritability, anger rumination, and self‐control were lagged to capture their potential effects on the following night's sleep. This method enables the assessment of temporal dynamics in emotion at the within‐person level. In addition, all models were adjusted for between‐person variables, assessed through baseline questionnaires, representing trait‐level characteristics (i.e., sleep quality and quantity, irritability, ER, and self‐control) (Supporting Information S1: Tables S1 and S2). Thus, between‐person differences were represented by both baseline trait measures and by person‐level averages derived from ESM, whereas within‐person variables (i.e., daily ESM assessments) reflected state‐level fluctuations.

Due to the hierarchical structure of the data with repeated measures, mixed‐effects models were used, with random intercept included for each participant. Two mixed‐effects models were fitted: (1) sleep quality served as the outcome variable, predicted by the lag of daily irritability's interaction with the lag of daily anger rumination and the lag of daily self‐control; (2) daily irritability was the outcome variable, with the lag of sleep quality, daily anger rumination, and daily self‐control as predictors. Random slopes for theoretically relevant within‐person predictors were tested for each model (Supporting Information S1: Tables S3–S8). Model comparisons using likelihood ratio tests and Akaike Information Criterion indicated that random‐intercept‐only models provided the best fit; therefore, these models were retained for all analyses. All models were fitted using the lme4 package in R, with *p*‐values for fixed effects derived from the lmerTest package using Satterthwaite degrees‐of‐freedom approximations.

To examine moderation effects, interaction terms were specified using the between‐person components of anger rumination and self‐control (i.e., person‐level means derived from ESM), rather than their within‐person deviations. This decision was theoretically driven, as anger rumination and self‐control are conceptualized as relatively stable individual tendencies that reflect a person's dispositional resource or personality trait (Denson, 2013; Paschke et al., 2016). Accordingly, we tested whether stable differences in anger rumination and self‐control moderated the within‐person association between daily sleep (within‐person fluctuations) and next‐day irritability. Thus, moderation was modeled as a cross‐level interaction (e.g., anger rumination [bp] × sleep [wp] and self‐control [bp] × sleep [bp] predicting irritability).

In case of a significant interaction, since the moderators are continuous variables, their moderating effects were tested at the median (moderate level), median + 1 SD (high level), and median − 1 SD (low level) for the sake of interpretability of the results.

Diagnostic plots were used to confirm compliance with model assumptions, including homoscedasticity and normality of residuals, and to check for influential outliers. The marginal and conditional *R* ^2^ values are reported as measures of model fit, representing the proportion of variance in the outcome explained by the fixed effects alone and by both the fixed and random effects, respectively. Additionally, the intraclass correlation coefficient is reported for each model to quantify the share of within‐person variability, estimated as the ratio of between‐person variance to the total variance. Statistical significance level was set at *p* ≤ 0.05. We used the R software environment (version 4.3.2) to compute statistics (79). Given the relatively modest sample size and the number of interaction terms examined, analyses should be considered exploratory. Therefore, formal corrections for multiple testing were not applied. Accordingly, results are interpreted with caution, with an emphasis on effect sizes rather than strict hypothesis testing.

## RESULTS

Table 1 reports the descriptive statistics.

Among the sample, participants were predominantly Swiss. Most spoke French as their first language, were still in school, and lived with their parents. Regarding compliance, all 56 participants completed the baseline questionnaires, and a total of 2016 ESM assessments were collected, with a mean response rate of 91.9% (median = 94.4%, ranging from 55.6% to 100%).

### Correlations

The coefficients of correlation are described in Table 2 and were computed exclusively from variables measured at baseline (i.e., trait‐level), thus reflecting static processes rather than within‐person temporal dynamics. At the between‐person level, low trait self‐control was associated with a greater use of trait maladaptive ER strategies (*r* = −0.27, *p* = .047) and higher trait irritability (*r* = −0.27, *p* = .042). Trait irritability was positively associated with trait maladaptive ER strategies (*r* = 0.26, *p* = .048). Additionally, fewer hours of sleep (sleep quantity) were associated with greater trait sleep difficulties (sleep quality) (*r* = −0.38, *p* = .004).

**TABLE 2:** Spearman correlation matrix for between‐person (upper part) based on baseline self‐report questionnaires and within‐person (lower part) variables using ambulatory assessment (*n* = 56).

Between‐person (trait)				
Variables	1. Trait maladaptive ER	2. Trait sleep difficulties	3. Trait sleep quantity	4. Trait irritability
1. Trait maladaptive ER	‐			
2. Trait sleep difficulties	0.10	‐		
3. Trait sleep quantity	−0.21	−0.38^∗∗^	‐	
4. Trait irritability	0.26^∗^	0.24	−0.18	‐
5. Trait self‐control	−0.27^∗^	−0.04	0.20	−0.28^∗^

Within‐person (state)			
1. Daily self‐control	1. Daily self‐control	2. Daily sleep quality	3. Daily irritability
2. Daily sleep quality	0.16^∗∗^	‐	‐
3. Daily irritability	−0.18	−0.11^∗^	‐
4. Daily anger rumination	−0.02	−0.013^∗∗∗^	0.27

Abbreviation: ER, emotion regulation.

^∗^ *p* < .05.

^∗∗^ *p* < .01.

^∗∗∗^ *p* < .001.

### Linear mixed effect models

Figure 1 illustrates the summary of the results for the irritability‐sleep and sleep‐irritability associations.

**FIGURE 1:**
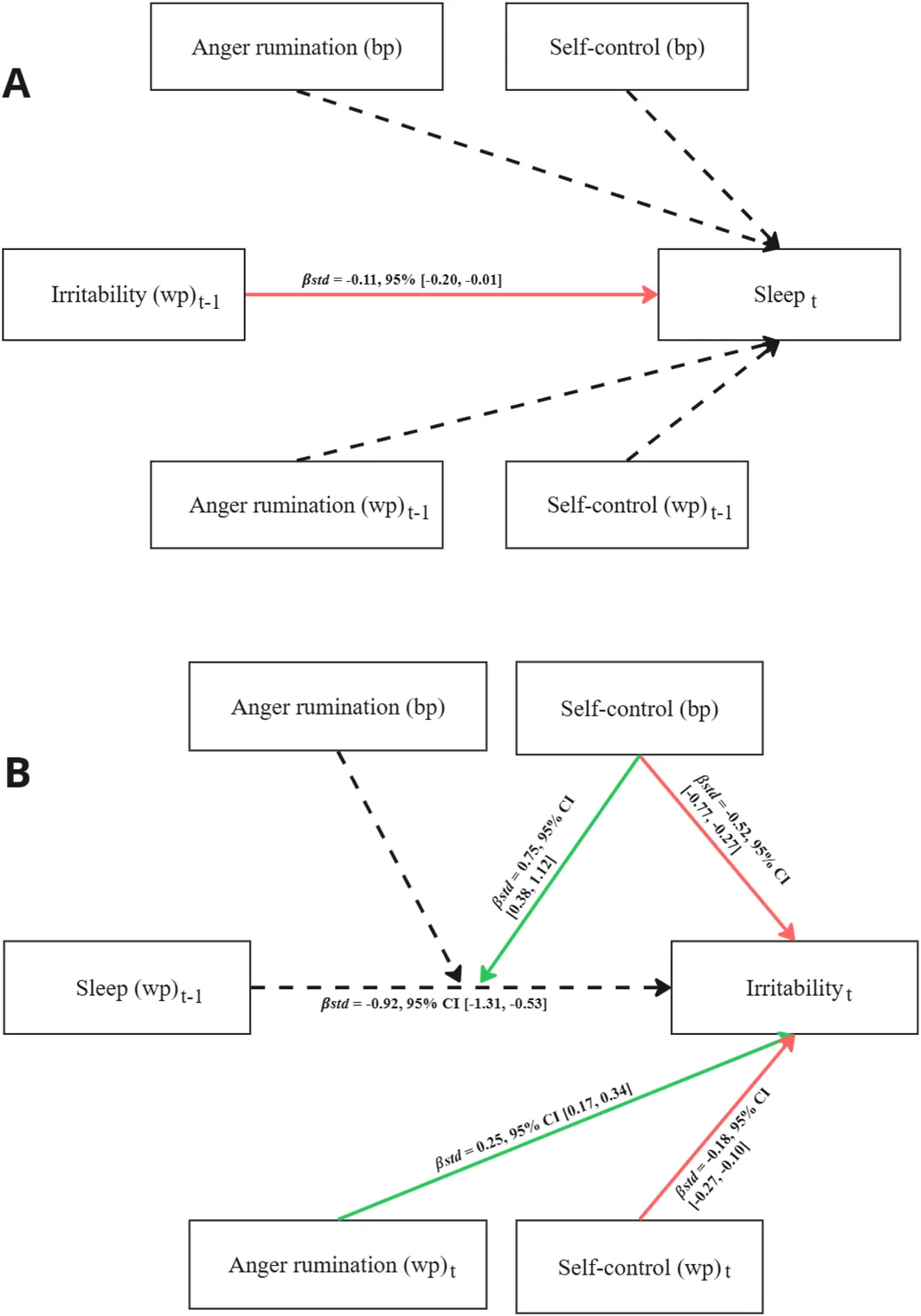
Overview of the main study findings. Panel (A) depicts the association between daytime irritability (measured at time −1) and sleep (measured the following morning at time *t*), with self‐control and anger rumination as moderators. Panel (B) illustrates the association between sleep (at time −1) and irritability assessed on the following day (time *t*), with both self‐control and anger rumination as moderators. Associations shown in green are positive, whereas those shown in red are negative. Dotted arrows represent hypothesized associations. Trait variables were assessed at baseline. bp, between‐person differences (person‐level means of ESM variables); ESM, experience sampling method; wp, within‐person fluctuations (deviations from person‐level means) obtained using person‐mean centering.

#### Within‐person associations

The present results reflect within‐person processes using ESM variables. Based on the linear mixed effect model fitted on sleep as the dependent variable, an association with daily irritability (wp) of the previous day was observed (*β*std = −0.11, 95% [−0.20, −0.01]). More specifically, irritability was modestly associated with subsequent night of sleep (Table 3). The magnitude of this association was relatively small, suggesting limited practical relevance despite the observed association. Conditional *R* ^2^ shows that 42.6% of the variation in the outcome was explained by this model.

**TABLE 3:** Linear mixed effect model predicting sleep quality.

Predictors	Sleep				
	Beta	Std. Beta	CI	Std. CI	*p*
(Intercept)	2.69	0.00	−3.19–8.58	0.00–0.00	.369
Age	−0.01	−0.01	−0.22–0.20	−0.32–0.29	.940
Trait maladaptive ER	−0.01	−0.06	−0.04–0.02	−0.34–0.22	.665
Trait sleep difficulties	−0.19	−0.09	−0.77–0.40	−0.37–0.19	.535
Trait sleep quantity	0.10	0.10	−0.24–0.43	−0.23–0.42	.562
Trait irritability	−0.38	−0.21	−0.93–0.16	−0.50–0.09	.165
Trait self‐control	0.01	0.10	−0.02–0.05	−0.16–0.37	.441
Anger rumination (bp)	−0.01	−0.10	−0.03–0.01	−0.39–0.19	.495
Self‐control (bp)	0.02	0.30	−0.00–0.04	−0.03–0.62	.075
Irritability (bp)	−0.02	−0.17	−0.06–0.02	−0.54–0.20	.364
Lag anger rumination (wp)	−0.00	−0.01	−0.01–0.01	−0.11–0.08	.798
Lag irritability (wp)	−0.01	−0.11	−0.02–−0.00	−0.20–−0.01	**.033**
Lag self‐control (wp)	0.00	0.02	−0.01–0.01	−0.07–0.11	.680
**Random effects**					
*σ* ^2^				1.54	
*τ*_00 id_task_				0.59	
*N*_id_task_				56	
Observations				462	
Marginal *R* ^2^/Conditional *R* ^2^				0.208/0.426	

*Note*: Values in bold means significant results. Trait variables were assessed at baseline.

Abbreviations: bp, between‐person differences (person‐level means of ESM variables); CI, 95% confidence interval; ER, emotion regulation; wp, within‐person fluctuations (deviations from person‐level means) obtained using person‐mean centering.

The mixed effect model fitted on daily irritability as the dependent variable is presented in Table 4. Sleep quality (wp) (*β*std = −0.92, 95% CI [−1.31, −0.53]) was related to daily irritability. Furthermore, two direct associations were found between daily self‐control (wp), daily anger rumination (wp) and daily irritability, with high levels of self‐control associated with decreased levels of irritability (*β*std = −0.18, 95% CI [−0.27, −0.10]) and high levels of anger rumination associated with higher levels of irritability (*β*std = 0.25, 95% CI [0.17, 0.34]).

**TABLE 4:** Linear mixed effect model predicting daily irritability.

Predictors	Daily irritability				
	Beta	Std. Beta	CI	Std. CI	*p*
(Intercept)	45.09	0.00	6.17–84.01	0.00–0.00	**.023**
Age	−1.58	−0.30	−2.95–−0.21	−0.55–−0.04	**.024**
Trait maladaptive ER	0.28	0.32	0.08–0.48	0.09–0.55	**.007**
Trait sleep difficulties	0.79	0.05	−3.24–4.83	−0.20–0.29	.699
Trait sleep quantity	−0.82	−0.10	−3.12–1.47	−0.39–0.18	.481
Trait irritability	2.94	0.20	−0.80–6.69	−0.06–0.46	.123
Trait self‐control	0.13	0.12	−0.12–0.38	−0.11–0.35	.316
Anger rumination (bp)	0.09	0.16	−0.05–0.23	−0.09–0.42	.197
Self‐control (bp)	−0.26	−0.52	−0.38–−0.13	−0.77–−0.27	**<.001**
Sleep (bp)	−0.77	−0.11	−2.74–1.21	−0.38–0.17	.447
Anger rumination (wp)	0.26	0.25	0.17–0.35	0.17–0.34	**<.001**
Self‐control (wp)	−0.24	−0.18	−0.35–−0.12	−0.27–−0.10	**<.001**
Sleep (wp)	−9.37	−0.92	−13.36–−5.38	−1.31–−0.53	**<.001**
Anger rumination (bp) × sleep (wp)	0.11	0.18	0.04–0.18	0.07–0.30	**.002**
Self‐control (bp) × sleep (wp)	0.10	0.75	0.05–0.15	0.38–1.12	**<.001**
**Random effects**					
*σ* ^2^				122.10	
*τ*_00id_task_				21.84	
*N*_id_task_				56	
Observations				465	
Marginal *R* ^2^/Conditional *R* ^2^				0.319/0.42	

*Note*: Values in bold means significant results. Trait variables were assessed at baseline.

Abbreviations: bp, between‐person differences (person‐level means of ESM variables); CI, 95% confidence interval; ER, emotion regulation; wp, within‐person fluctuations (deviations from person‐level means) obtained using person‐mean centering.

#### Between‐person associations

Age at baseline was negatively associated with daily irritability, indicating lower levels of daily irritability with increasing age (*β*std = −0.30, 95% [−0.55, −0.04]). Importantly, controlling for age did not substantially alter the pattern of the main predictors (Supporting Information S1: Table S9). The baseline trait variable maladaptive ER strategy was associated with daily irritability assessed through ESM (*β*std = 0.32, 95% CI [0.09, 0.55]), whereas other trait variables such as trait sleep difficulties, trait sleep quantity, trait irritability, and trait self‐control, assessed at baseline, were not. However, between‐person differences in self‐control derived from ESM person‐means were negatively associated with daily irritability (*β*std = −0.52, 95% CI [−0.77, −0.27]).

#### Cross‐level interactions

An interaction between between‐person level anger rumination (bp) and within‐person sleep quality (wp) on daily irritability was observed (*β*std_interaction_ = 0.18, 95% CI [0.07, 0.30]). Simple slope analyses indicated that among individuals with low anger rumination (−1 SD below the mean), higher sleep quality was associated with lower daily irritability. In contrast, among adolescents with average (mean) and high anger rumination (+1 SD above the mean), the association between sleep quality and irritability was attenuated and no longer substantial (Figure 2).

**FIGURE 2:**
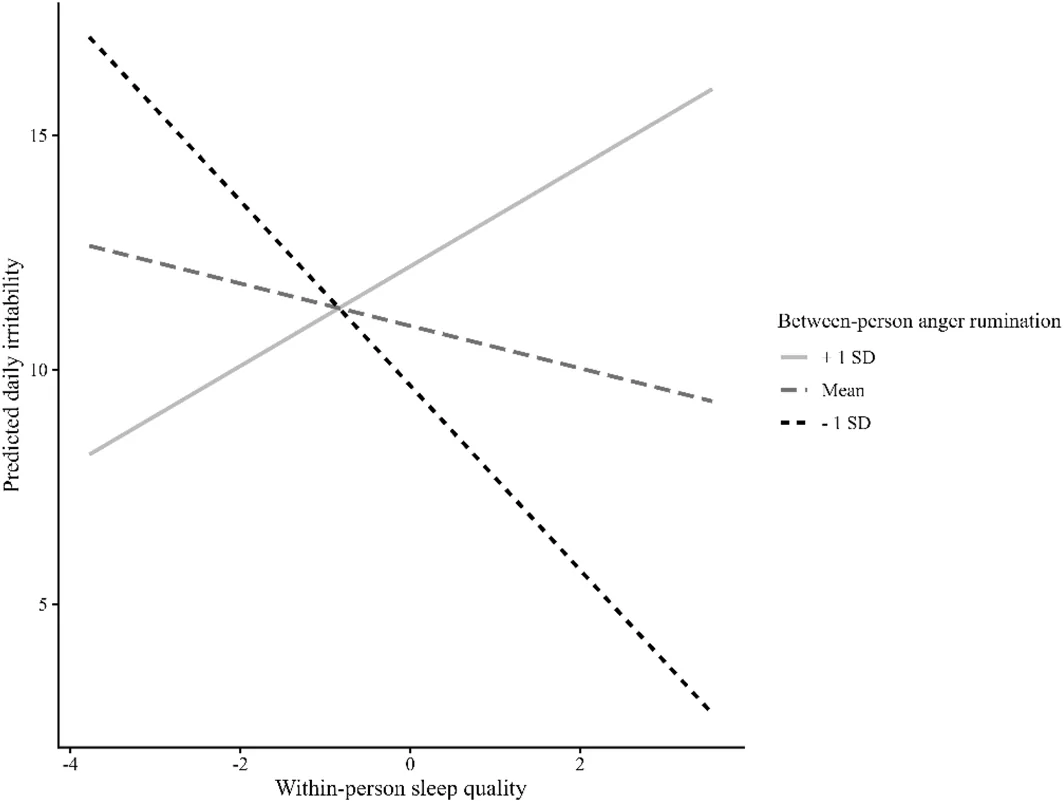
Moderating effect of anger rumination (bp) on the relationship between daily sleep quality and daily irritability. Lines represent predicted values derived from the fitted mixed‐effects model based on simple slopes analyses. Slopes were estimated using simple slopes analyses at ±1 SD of the moderator. bp, between‐person differences (person‐level mean of ESM variable); ESM, experience sampling method.

The interaction was also found between self‐control (bp), daily sleep quality (wp) and daily irritability (*β*std_interaction_ = 0.75, 95% CI [0.38, 1.12]), indicating that the association between sleep quality and irritability differed depending on self‐control levels between adolescents. Specifically, simple slope analyses showed that individuals with average to high levels of self‐control (mean and +1 SD above the mean), exhibited non‐substantial reductions in levels of irritability irrespective of high sleep quality. By contrast, in individuals with lower levels of self‐control (−1 SD below the mean), better sleep quality was associated with lower levels of irritability (Figure 3).

**FIGURE 3:**
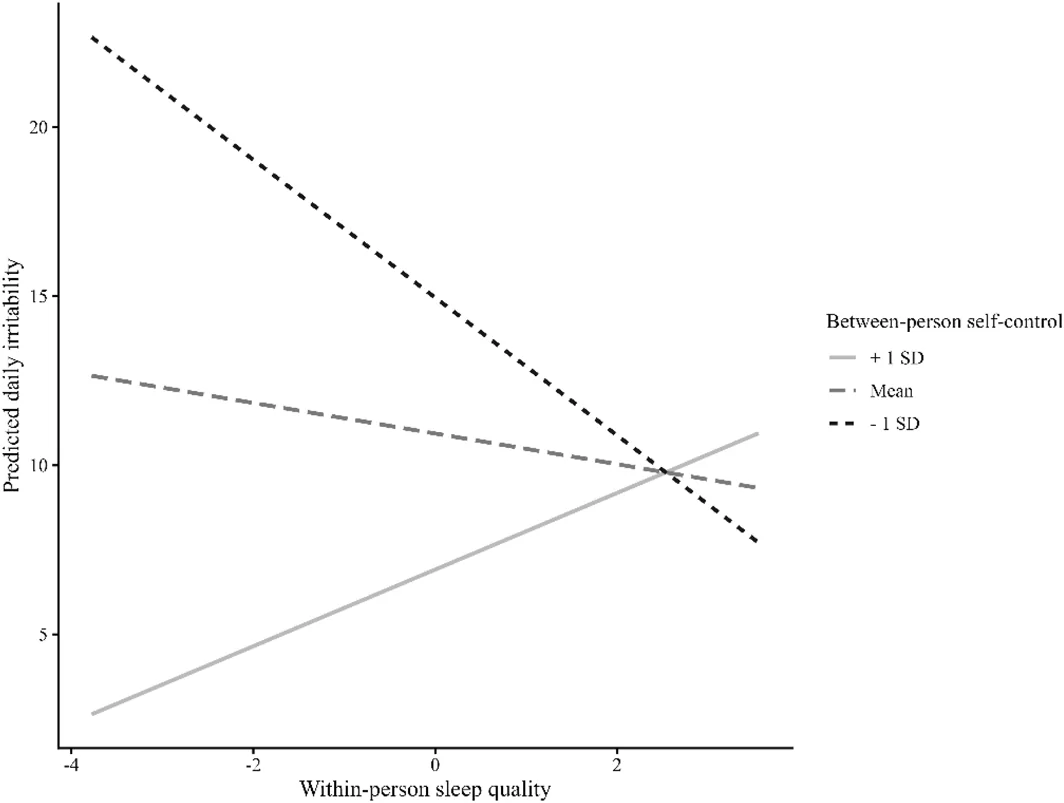
Moderating effect of self‐control (bp) on the relationship between daily sleep quality and daily irritability. Lines represent predicted values derived from the fitted mixed‐effects model based on simple slopes analyses. Slopes were estimated using simple slopes analyses at ±1 SD of the moderator. bp, between‐person differences (person‐level mean of ESM variable); ESM, experience sampling method.

## DISCUSSION

The present study investigated the day‐to‐day associations between sleep and irritability in a sample of male adolescents, considering daily anger rumination and self‐control as potential moderators. Overall, findings indicated that sleep quality was directly associated with daily irritability. Additionally, the effect of this association might vary according to the between‐person difference in anger rumination and self‐control.

Furthermore, anger rumination and self‐control at the within‐person level were directly associated with daily irritability. In the reverse association, irritability from the day on subsequent night of sleep was observed, although size effects were small.

This exploratory study centered its analysis on irritability in male adolescents exclusively. It contributes to the literature by offering preliminary insights into how sleep operates as a recovery process for ER as well as for cognitive resources.

### Associations at the within‐person level

At the within‐person level using ESM measures, results showed a relationship between sleep quality and daily irritability, thereby aligning with existing literature suggesting that both constructs may be linked in daily life (Meigs et al., 2024). However, findings should be interpreted cautiously due to the small effect size and the exploratory nature of the analysis. Additionally, between‐person differences in anger rumination and self‐control were associated with variation in the strength of the sleep‐irritability relationship.

Specifically, the association between sleep quality and next‐day irritability was stronger among adolescents with lower levels of anger rumination and weaker among those with moderate to high levels of anger rumination. In particular, at moderate to high daily levels of anger rumination, the magnitude of the sleep‐irritability association was substantially smaller. Theoretically, it has been proposed that individuals who engage in repetitive and persistent thoughts about anger‐inducing events experienced disrupted sleep, potentially interfering with processes that have been proposed to support emotional recovery during sleep (Li et al., 2019; Vandekerckhove & Wang, 2018). Our preliminary findings may be interpreted in light of the emotional cascade theory, which proposes an exponential negative feedback loop effect that may impair adolescents' functioning across academic, familial, and social domains (Selby et al., 2016). In addition, a direct association was observed between anger rumination during the day and next‐day irritability. Anger rumination has been described as the tendency to mentally rehearse anger‐provoking events, including their causes and their consequences, rather than disengaging from them. Prior ESM studies have shown that anger rumination co‐occurs with negative affect and may predict worse mood later, which, in the present context, be reflected in irritability (Boemo et al., 2022; Kircanski, Thompson, et al., 2018).

Regarding self‐control, the association between sleep quality and next‐day irritability was stronger at lower levels of daily self‐control, such that higher sleep quality was related to lower daily levels of irritability. In contrast, this association was weaker at moderate to high levels of self‐control. This pattern might suggest that the association between sleep quality and irritability differs across levels of self‐control. Specifically, this association appeared weaker at higher levels of self‐control, suggesting that irritability may be less sensitive to variations in sleep in these adolescents. We also observed a direct association between self‐control from the day and next‐day irritability.

In addition, age was negatively associated with daily irritability. This finding is consistent with the literature suggesting that, during adolescence, irritability diverges into distinct developmental trajectories, representing a critical period for emotional development (Leibenluft et al., 2024). The negative association in the present study may therefore reflect normative developmental changes in irritability across this age period.

Interestingly, we observed an association between levels of irritability during the day and sleep quality assessed the subsequent morning, which is consistent with findings in existing literature (Meigs et al., 2024). However, findings should be interpreted cautiously, as they are preliminary and require replication.

### Associations at the between‐person level

At the between‐person level, using baseline measures, results indicated that adolescents reporting greater trait sleep difficulties also reported reduced hours of sleep. In addition, adolescents with higher trait irritability reported greater use of trait maladaptive ER strategies and higher levels of trait self‐control. These results are consistent with previous research showing that sleep disorders (e.g., insomnia) tend to co‐occur with a range of negative consequences, including tiredness, mood instability, impaired daytime functioning, slower cognitive responses, and increased consumption of stimulants such as caffeine or alcohol (Nelson et al., 2022). However, given the correlational nature of these findings, the observed associations should not be interpreted as evidence of causal effects.

### Clinical implications

Finally, the findings of this exploratory study highlight the complexity of the relationship between sleep and irritability in adolescent males. Indeed, our results suggest that an association may exist between these two constructs. In addition, in the context of our study, this relationship appears to be influenced by rumination and self‐control. Regarding emotion dysregulation, better use of adaptive ER strategies could potentially enhance self‐control skills and improve coping mechanisms in anger‐related events, which may decrease anger rumination. In parallel, improving inhibition abilities may be related to sleep‐related outcomes, although the present findings do not allow conclusions regarding the mechanistic processes linking these constructs. For instance, redirecting attention when confronted with an immediate potential reward or using mental reframing are strategies that strengthen self‐control skills (Fujita et al., 2020). Nevertheless, from both a preventive perspective and in light of the existing literature, it remains important to consider sleep‐focused interventions directly, as improving sleep may help prevent the emergence or maintenance of irritability. Indeed, targeted interventions such as sleep education, reducing screen use before bedtime, promoting physical activity, limiting caffeine consumption, and establishing bedtime rituals could positively impact mental well‐being in youths (Johri et al., 2025; Snel & Lorist, 2011).

In the opposite direction, our findings suggest that irritability may influence sleep, highlighting the potential bidirectional nature of this relationship. This highlights irritability as a potentially relevant target for future interventions aimed at improving sleep outcomes. For instance, a novel exposure‐based cognitive‐behavioral therapy that exposes adolescents to frustration‐inducing situations, combined with parent management training aimed at reducing the reinforcement of irritable behaviors, might help improve ER skills (Kircanski, Clayton, et al., 2018; Kircanski et al., 2019; Linke et al., 2020). Nevertheless, these preliminary findings should be investigated more thoroughly in future research.

### Limitations

While the current study was the first to examine relationships between irritability, sleep, anger rumination, and self‐control, several limitations merit consideration. First, the sample size was small, particularly for testing multiple cross‐level interactions. While intensive longitudinal ESM designs increase statistical power by leveraging repeated within‐person observations, they do not fully eliminate the risk of Type I error. Cross‐level effects should therefore be interpreted with caution and considered exploratory pending replication in larger samples. Additionally, it was uniquely composed of male adolescents, therefore it limits the generalizability of the findings. Accordingly, the conclusions should be interpreted as specific to adolescent males and may not extend to girls or to adolescents more broadly. Further studies should involve larger samples composed of female and male adolescents, since sex differences have been found in ER processes (Nolen‐Hoeksema, 2012). Second, we focused on two specific components in the day‐to‐day associations between sleep and irritability. Other constructs such as impulsivity, chronotype, daytime sleepiness, and physiological biomarkers (e.g., heart rate variability, cortisol level) might be relevant to include in future research. Similarly, we only examined anger rumination as a maladaptive ER strategy and self‐control as an adaptive one, which limits the ability to capture the full spectrum of ER processes. Third, although this study examined sleep quality based on participants’ subjective assessments, incorporating objective measures could help reach optimal reliability. Moreover, the baseline measure of trait sleep difficulties and irritability and the ESM measure of self‐control showed low internal consistency. This modest reliability may have introduced increased measurement error, thereby attenuating interaction effects and reducing confidence in the moderation findings. Additionally, sleep duration may not be a reliable indicator of sleep quantity, as it naturally fluctuates within individuals across nights. In the present study, it was assessed as a single between‐person measure, which may fail to capture this intra‐individual variability and should therefore be interpreted with caution. Although previous research has demonstrated high convergent validity between YSR sleep items and measures such as actigraphy and sleep diaries (Gregory et al., 2011), our findings suggest that future studies may benefit from including validated sleep questionnaires and/or objective measures to better capture adolescents' habitual sleep patterns. Fourth, the ESM item used to assess irritability in the present study did not explicitly reference irritability terminology and may reflect anger, distress or frustration more generally. While these constructs can be summarized as irritability (Saatchi et al., 2023; Tseng et al., 2023), our findings should be interpreted accordingly. Additionally, to better control for confounding variables, measures of sleep disorders such as insomnia, apnea, or other disturbances likely to modulate sleep patterns would have been relevant to include at baseline. Similarly, the occurrence of naps and weekend assessments were not considered in the ambulatory assessment, which might also limit generalization and influence adolescents' levels of rest, respectively. Finally, future studies should include evening‐specific assessments of irritability, given that emotional states occurring near bedtime may exert a more immediate influence on sleep initiation and sleep quality (Harvey, 2002).

### Future perspectives

Future research should further explore the long‐term reciprocal effects of sleep and irritability across different developmental stages (i.e., childhood) and among girls, examining the existing psychopathological trajectories. Moreover, exploring the brain mechanisms underlying the interplay between irritability, sleep, self‐control, and anger rumination could provide better insights into ER processes. Regarding sleep quality assessment, considering objective measures including actigraphy could enable a more detailed differentiation of sleep parameters and their influence on ER. Finally, given the conceptual overlap between irritability and the anger‐related dimensions mentioned previously, future studies should aim to disentangle their shared and distinct emotional and cognitive components, as well as to develop accurate and validated scales for their assessment and distinction (Zik et al., 2022).

## CONCLUSION

To conclude, our preliminary findings suggest that the association between sleep and next‐day irritability may exist and that this relationship may vary as a function of anger rumination and self‐control at the between‐person level. However, given the exploratory nature of the current study and the modest sample size, findings require replication.

In the reverse direction, namely the effect of daytime irritability on subsequent night of sleep, an association was also observed, although the effect size was relatively modest. Notably, the association between sleep and subsequent irritability appeared larger in magnitude than the association between irritability and subsequent sleep. Ultimately, these effects should be interpreted with caution since the underlying mechanisms cannot be determined from the present data.

Overall, the current study offers exploratory insights into the day‐to‐day temporal associations among irritability, sleep, anger rumination, and self‐control in a sample of male adolescents.

## AUTHOR CONTRIBUTIONS

**Julia Giovannini**: Formal analysis; methodology; writing—original draft preparation; writing—review and editing; **Setareh Ranjbar**: Formal analysis; writing—review and editing. **Jennifer Glaus**: preliminary analysis; writing—review and editing. **Ines Mürner‐Lavanchy**: Writing—review and editing. **Lauriane Constanty**: Investigation; data curation; writing—review and editing. **Kerstin Jessica Plessen**: Conceptualization; methodology; writing—review and editing. **Sébastien Urben**: Conceptualization; funding acquisition; writing—review and editing.

## CONFLICT OF INTEREST STATEMENT

The authors declare no conflicts of interest.

## ETHICAL CONSIDERATIONS

The study was approved by the Cantonal Research Ethics Commission of Vaud (CER‐VD) under the reference number 2019‐02318 on 8 June 2020 and was conducted in accordance with the Declaration of Helsinki. Written informed consent was obtained from all participants.

## Supporting information

Supporting Information S1

## Data Availability

The data that support the findings of this study are available from the corresponding author upon reasonable request.

## References

[jcv270183-bib-0001] Aldao, A. , & Nolen‐Hoeksema, S. (2012). The influence of context on the implementation of adaptive emotion regulation strategies. Behaviour Research and Therapy, 50(7–8), 493–501. 10.1016/j.brat.2012.04.004 22659159

[jcv270183-bib-0002] Alloy, L. B. , Hamilton, J. L. , Hamlat, E. J. , & Abramson, L. Y. (2016). Pubertal development, emotion regulatory styles, and the emergence of sex differences in internalizing disorders and symptoms in adolescence. Clinical Psychological Science, 4(5), 867–881. 10.1177/2167702616643008 PMC5061504 27747141

[jcv270183-bib-0003] American Psychiatric Association . (2013). Diagnostic and statistical manual of mental disorders (5 ed.). American Psychiatric Publishing.

[jcv270183-bib-0004] Anastasiades, P. G. , de Vivo, L. , Bellesi, M. , & Jones, M. W. (2022). Adolescent sleep and the foundations of prefrontal cortical development and dysfunction. Progress in Neurobiology, 218, 102338. 10.1016/j.pneurobio.2022.102338 PMC7616212 35963360

[jcv270183-bib-0005] Baum, K. T. , Desai, A. , Field, J. , Miller, L. E. , Rausch, J. , & Beebe, D. W. (2014). Sleep restriction worsens mood and emotion regulation in adolescents. Journal of Child Psychology and Psychiatry, 55(2), 180–190. 10.1111/jcpp.12125 PMC4047523 24889207

[jcv270183-bib-0006] Bell, E. , Boyce, P. , Porter, R. J. , Bryant, R. A. , & Malhi, G. S. (2021). Irritability in mood disorders: Neurobiological underpinnings and implications for pharmacological intervention. CNS Drugs, 35(6), 619–641. 10.1007/s40263-021-00823-y 34019255

[jcv270183-bib-0007] Boemo, T. , Nieto, I. , Vazquez, C. , & Sanchez‐Lopez, A. (2022). Relations between emotion regulation strategies and affect in daily life: A systematic review and meta‐analysis of studies using ecological momentary assessments. Neuroscience & Biobehavioral Reviews, 139, 104747. 10.1016/j.neubiorev.2022.104747 35716875

[jcv270183-bib-0008] Boon, M. E. , van Hooff, M. L. M. , Vink, J. M. , & Geurts, S. A. E. (2023). The effect of fragmented sleep on emotion regulation ability and usage. Cognition & Emotion, 37(6), 1132–1143. 10.1080/02699931.2023.2224957 37337975

[jcv270183-bib-0009] Brevers, D. , Foucart, J. , Verbanck, P. , & Turel, O. (2017). Examination of the validity and reliability of the French version of the Brief Self‐Control Scale. Canadian Journal of Behavioural Science, 49(4), 243–250. 10.1037/cbs0000086 PMC5707128 29200467

[jcv270183-bib-0010] Carskadon, M. A. (2011). Sleep in adolescents: The perfect storm. Pediatric Clinics of North America, 58(3), 637–647. 10.1016/j.pcl.2011.03.003 PMC3130594 21600346

[jcv270183-bib-0011] Chad‐Friedman, E. , Galano, M. M. , Lemay, E. P. , Olino, T. M. , Klein, D. N. , & Dougherty, L. R. (2021). Parsing between‐ and within‐person effects: Longitudinal associations between irritability and internalizing and externalizing problems from early childhood through adolescence. Development and Psychopathology, 35(3), 1371–1381. 10.1017/S0954579421001267 PMC9234095 34955108

[jcv270183-bib-0012] Chattu, V. K. , Manzar, M. D. , Kumary, S. , Burman, D. , Spence, D. W. , & Pandi‐Perumal, S. R. (2018). The global problem of insufficient sleep and its serious public health implications. Healthcare (Basel), 7(1), 1. 10.3390/healthcare7010001 PMC6473877 30577441

[jcv270183-bib-0013] Crowley, S. J. , Wolfson, A. R. , Tarokh, L. , & Carskadon, M. A. (2018). An update on adolescent sleep: New evidence informing the perfect storm model. Journal of Adolescence, 67(1), 55–65. 10.1016/j.adolescence.2018.06.001 PMC6054480 29908393

[jcv270183-bib-0014] d’Acremont, M. , & Van der Linden, M. (2007). How is impulsivity related to depression in adolescence? Evidence from a French validation of the Cognitive Emotion Regulation Questionnaire. Journal of Adolescence, 30(2), 271–282. 10.1016/j.adolescence.2006.02.007 16600359

[jcv270183-bib-0015] Delahoyde, M. K. , Tyack, C. , Kugarajah, S. , & Joseph, D. (2024). Insomnia and other sleep disorders in adolescence. BMJ Paediatrics Open, 8(1), e001229. 10.1136/bmjpo-2021-001229 PMC11293389 39084851

[jcv270183-bib-0016] Denson, T. F. (2013). The multiple systems model of angry rumination. Personality and Social Psychology Review, 17(2), 103–123. 10.1177/1088868312467086 23175519

[jcv270183-bib-0017] Denson, T. F. , DeWall, C. N. , & Finkel, E. J. (2012). Self‐control and aggression. Current Directions in Psychological Science, 21(1), 20–25. 10.1177/0963721411429451

[jcv270183-bib-0018] Derella, O. J. , Johnston, O. G. , Loeber, R. , & Burke, J. D. (2019). CBT‐enhanced emotion regulation as a mechanism of improvement for childhood irritability. Journal of Clinical Child and Adolescent Psychology, 48(sup1), S146–S154. 10.1080/15374416.2016.1270832 PMC6283701 28151019

[jcv270183-bib-0019] Diamond, A. (2013). Executive functions. Annual Review of Psychology, 64(1), 135–168. 10.1146/annurev-psych-113011-143750 PMC4084861 23020641

[jcv270183-bib-0020] Dougherty, L. R. , Galano, M. M. , Chad‐Friedman, E. , Olino, T. M. , Bufferd, S. J. , & Klein, D. N. (2021). Using item response theory to compare irritability measures in early adolescent and childhood samples. Assessment, 28(3), 918–927. 10.1177/1073191120936363 PMC8724905 32613838

[jcv270183-bib-0021] Duston, A. , Holtman, S. , Bowen, A. E. , Cree, M. G. , Nadeau, K. , Wright, K. P., Jr. , Simon, S. L. , & Diniz Behn, C. G. (2025). Sex differences in circadian timing and biological night in adolescents. Journal of Biological Rhythms, 40(1), 7–18. 10.1177/07487304241309165 PMC12285599 39876068

[jcv270183-bib-0022] Ellis, L. K. , & Rothbart, M. K. (2001). Revision of the early adolescent temperament questionnaire. Bilennial Meeting of the Society for Research in Child Development.

[jcv270183-bib-0023] Evans, S. C. , Bonadio, F. T. , Bearman, S. K. , Ugueto, A. M. , Chorpita, B. F. , & Weisz, J. R. (2020). Assessing the irritable and defiant dimensions of youth oppositional behavior using CBCL and YSR items. Journal of Clinical Child and Adolescent Psychology, 49(6), 804–819. 10.1080/15374416.2019.1622119 31276433

[jcv270183-bib-0024] Evans, S. C. , Corteselli, K. A. , Edelman, A. , Scott, H. , & Weisz, J. R. (2023). Is irritability a top problem in youth mental health care? A multi‐informant, multi‐method investigation. Child Psychiatry and Human Development, 54(4), 1027–1041. 10.1007/s10578-021-01301-8 35064392

[jcv270183-bib-0025] Fernandez, A. V. (2019). Phenomenology and dimensional approaches to psychiatric research and classification. Philosophy, Psychiatry, and Psychology, 26(1), 65–75. 10.1353/ppp.2019.0004

[jcv270183-bib-0026] Ferrari, J. R. , Stevens, E. B. , & Jason, L. A. (2009). The role of self‐regulation in abstinence maintenance: Effects of communal living on self‐regulation. Journal of Groups in Addiction & Recovery, 4(1–2), 32–41. 10.1080/15560350802712371 PMC2916178 20689650

[jcv270183-bib-0027] Fujita, K. , Orvell, A. , & Kross, E. (2020). Smarter, not harder: A toolbox approach to enhancing self‐control. Policy Insights from the Behavioral and Brain Sciences, 7(2), 149–156. 10.1177/2372732220941242

[jcv270183-bib-0028] Garnefski, N. , Kraaij, V. , & Spinhoven, P. (2001). Negative life events, cognitive emotion regulation and emotional problems. Personality and Individual Differences, 30(8), 1311–1327. 10.1016/S0191-8869(00)00113-6

[jcv270183-bib-0029] Gregory, A. M. , Cousins, J. C. , Forbes, E. E. , Trubnick, L. , Ryan, N. D. , Axelson, D. A. , Birmaher, B. , Sadeh, A. , & Dahl, R. E. (2011). Sleep items in the child behavior checklist: A comparison with sleep diaries, actigraphy, and polysomnography. Journal of the American Academy of Child & Adolescent Psychiatry, 50(5), 499–507. 10.1016/j.jaac.2011.02.003 PMC3581333 21515199

[jcv270183-bib-0030] Grondal, M. , Ask, K. , & Winblad, S. (2023). The distinction between irritability and anger and their associations with impulsivity and subjective wellbeing. Scientific Reports, 13(1), 10398. 10.1038/s41598-023-37557-4 PMC10300089 37369760

[jcv270183-bib-0031] Hagger, M. S. (2010). Sleep, self‐regulation, self‐control and health. Stress and Health, 26(3), 181–185. 10.1002/smi.1345

[jcv270183-bib-0032] Harvey, A. G. (2002). A cognitive model of insomnia. Behaviour Research and Therapy, 40(8), 869–893. 10.1016/s0005-7967(01)00061-4 12186352

[jcv270183-bib-0033] Houben, M. , Claes, L. , Vansteelandt, K. , Berens, A. , Sleuwaegen, E. , & Kuppens, P. (2017). The emotion regulation function of nonsuicidal self‐injury: A momentary assessment study in inpatients with borderline personality disorder features. Journal of Abnormal Psychology, 126(1), 89–95. 10.1037/abn0000229 27808541

[jcv270183-bib-0034] Humphreys, K. L. , Schouboe, S. N. F. , Kircanski, K. , Leibenluft, E. , Stringaris, A. , & Gotlib, I. H. (2019). Irritability, externalizing, and internalizing psychopathology in adolescence: Cross‐sectional and longitudinal associations and moderation by sex. Journal of Clinical Child and Adolescent Psychology, 48(5), 781–789. 10.1080/15374416.2018.1460847 PMC6215733 29667523

[jcv270183-bib-0035] Jha, M. K. , Fava, M. , Minhajuddin, A. , Chin Fatt, C. , Mischoulon, D. , Wakhlu, N. , Trombello, J. M. , Cusin, C. , & Trivedi, M. H. (2021). Anger attacks are associated with persistently elevated irritability in MDD: Findings from the EMBARC study. Psychological Medicine, 51(8), 1355–1363. 10.1017/S0033291720000112 32138798

[jcv270183-bib-0036] Johri, K. , Pillai, R. , Kulkarni, A. , & Balkrishnan, R. (2025). Effects of sleep deprivation on the mental health of adolescents: A systematic review. Sleep Science and Practice, 9(1), 9. 10.1186/s41606-025-00127-w

[jcv270183-bib-0037] Kansagra, S. (2020). Sleep disorders in adolescents. Anales de Pediatía, 145(Suppl 2), S204–S209. 10.1542/peds.2019-2056I 32358212

[jcv270183-bib-0038] Kaufman, J. , Birmaher, B. , Brent, D. A. , Ryan, N. D. , & Rao, U. (2018). K‐SADS‐PL. Journal of the American Academy of Child & Adolescent Psychiatry, 39(10), 1208. 10.1097/00004583-200010000-00002 11026169

[jcv270183-bib-0039] Kessel, E. M. , Dougherty, L. R. , Hubacheck, S. , Chad‐Friedman, E. , Olino, T. , Carlson, G. A. , & Klein, D. N. (2021). Early predictors of adolescent irritability. Child and Adolescent Psychiatric Clinics of North America, 30(3), 475–490. 10.1016/j.chc.2021.04.002 PMC9004710 34053681

[jcv270183-bib-0040] Kim, J. , Noh, J. W. , Kim, A. , & Kwon, Y. D. (2022). The impact of weekday‐to‐weekend sleep differences on health outcomes among adolescent students. Children, 9(1), 52. 10.3390/children9010052 PMC8774225 35053677

[jcv270183-bib-0041] Kircanski, K. , Clayton, M. E. , Leibenluft, E. , & Brotman, M. A. (2018). Psychosocial treatment of irritability in youth. Current Treatment Options in Psychiatry, 5(1), 129–140. 10.1007/s40501-018-0141-5 PMC6181450 30319935

[jcv270183-bib-0042] Kircanski, K. , Craske, M. G. , Averbeck, B. B. , Pine, D. S. , Leibenluft, E. , & Brotman, M. A. (2019). Exposure therapy for pediatric irritability: Theory and potential mechanisms. Behaviour Research and Therapy, 118, 141–149. 10.1016/j.brat.2019.04.007 PMC6590706 31085355

[jcv270183-bib-0043] Kircanski, K. , Thompson, R. J. , Sorenson, J. , Sherdell, L. , & Gotlib, I. H. (2018). The everyday dynamics of rumination and worry: Precipitant events and affective consequences. Cognition & Emotion, 32(7), 1424–1436. 10.1080/02699931.2017.1278679 PMC6218316 28103761

[jcv270183-bib-0044] Klein, D. N. , Dougherty, L. R. , Kessel, E. M. , Silver, J. , & Carlson, G. A. (2021). A transdiagnostic perspective on youth irritability. Current Directions in Psychological Science, 30(5), 437–443. 10.1177/09637214211035101 PMC8765598 35046617

[jcv270183-bib-0045] Kraaij, V. , & Garnefski, N. (2012). Coping and depressive symptoms in adolescents with a chronic medical condition: A search for intervention targets. Journal of Adolescence, 35(6), 1593–1600. 10.1016/j.adolescence.2012.06.007 22771158

[jcv270183-bib-0046] Kuppens, P. , Oravecz, Z. , & Tuerlinckx, F. (2010). Feelings change: Accounting for individual differences in the temporal dynamics of affect. Journal of Personality and Social Psychology, 99(6), 1042–1060. 10.1037/a0020962 20853980

[jcv270183-bib-0047] Lazarus, P. J. , & Costa, A. (2020). Teaching emotional self‐regulation to children and adolescents. In P. J. Lazarus , S. Suldo , & B. Doll (Eds.), Fostering the emotional well‐being of our youth: A school‐based approach (pp. 264–281). Oxford University Press. 10.1093/med-psych/9780190918873.003.0014

[jcv270183-bib-0048] Leibenluft, E. , Allen, L. E. , Althoff, R. R. , Brotman, M. A. , Burke, J. D. , Carlson, G. A. , Dickstein, D. P. , Dougherty, L. R. , Evans, S. C. , Kircanski, K. , Klein, D. N. , Malone, E. P. , Mazefsky, C. A. , Nigg, J. , Perlman, S. B. , Pine, D. S. , Roy, A. K. , Salum, G. A. , Shakeshaft, A. , …, & Stringaris, A. (2024). Irritability in youths: A critical integrative review. American Journal of Psychiatry, 181(4), 275–290. 10.1176/appi.ajp.20230256 PMC12010774 38419494

[jcv270183-bib-0049] Leibenluft, E. , & Stoddard, J. (2013). The developmental psychopathology of irritability. Development and Psychopathology, 25(4 Pt 2), 1473–1487. 10.1017/S0954579413000722 PMC4476313 24342851

[jcv270183-bib-0050] Leigh, E. , Lee, A. , Brown, H. M. , Pisano, S. , & Stringaris, A. (2020). A prospective study of rumination and irritability in youth. Journal of Abnormal Child Psychology, 48(12), 1581–1589. 10.1007/s10802-020-00706-8 PMC7554009 33001331

[jcv270183-bib-0051] Levkovich, I. , Yatzkar, U. , & Shenaar‐Golan, V. (2025). Age and gender differences in emotional and behavioral functioning among youth referred to a Psychiatric Outpatient Clinic at a Public Hospital. Children (Basel), 12(6), 683. 10.3390/children12060683 PMC12191876 40564641

[jcv270183-bib-0052] Lewis, M. , Alessandri, S. M. , & Sullivan, M. W. (1990). Violation of expectancy, loss of control, and anger expressions in young infants. Developmental Psychology, 26(5), 745–751. 10.1037/0012-1649.26.5.745

[jcv270183-bib-0053] Li, Y. , Gu, S. , Wang, Z. , Li, H. , Xu, X. , Zhu, H. , Deng, S. , Ma, X. , Feng, G. , Wang, F. , & Huang, J. H. (2019). Relationship between stressful life events and sleep quality: Rumination as a mediator and resilience as a moderator. Frontiers in Psychiatry, 10, 348. 10.3389/fpsyt.2019.00348 PMC6545794 31191370

[jcv270183-bib-0054] Lievaart, M. , Huijding, J. , van der Veen, F. M. , Hovens, J. E. , & Franken, I. H. (2017). The impact of angry rumination on anger‐primed cognitive control. Journal of Behavior Therapy and Experimental Psychiatry, 54, 135–142. 10.1016/j.jbtep.2016.07.016 27494341

[jcv270183-bib-0055] Linke, J. , Kircanski, K. , Brooks, J. , Perhamus, G. , Gold, A. L. , & Brotman, M. A. (2020). Exposure‐based cognitive‐behavioral therapy for disruptive mood dysregulation disorder: An evidence‐based case study. Behavior Therapy, 51(2), 320–333. 10.1016/j.beth.2019.05.007 PMC9719109 32138941

[jcv270183-bib-0056] Liu, J. , Zhu, L. , & Liu, C. (2020). Sleep quality and self‐control: The mediating roles of positive and negative affects. Frontiers in Psychology, 11, 607548. 10.3389/fpsyg.2020.607548 PMC7773722 33391127

[jcv270183-bib-0057] Martel, M. M. (2013). Sexual selection and sex differences in the prevalence of childhood externalizing and adolescent internalizing disorders. Psychological Bulletin, 139(6), 1221–1259. 10.1037/a0032247 23627633

[jcv270183-bib-0058] Medicine, A. A. o. S. (2016). Recommended amount of sleep for pediatric populations: A consensus statement of the American Academy of Sleep Medicine. Journal of Clinical Sleep Medicine, 12(6), 785–786. 10.5664/jcsm.5866 PMC4877308 27250809

[jcv270183-bib-0059] Meigs, J. M. , Kiderman, M. , Kircanski, K. , Cardinale, E. M. , Pine, D. S. , Leibenluft, E. , Brotman, M. A. , & Naim, R. (2024). Sleepless nights, sour moods: Daily sleep‐irritability links in a pediatric clinical sample. Journal of Child Psychology and Psychiatry, 65(9), 1175–1183. 10.1111/jcpp.13959 PMC11322421 38355141

[jcv270183-bib-0060] Meltzer, L. J. (2017). Future directions in sleep and developmental psychopathology. Journal of Clinical Child and Adolescent Psychology, 46(2), 295–301. 10.1080/15374416.2016.1236727 27880041

[jcv270183-bib-0061] Myin‐Germeys, I. , & Kuppens, P. (2022). The open handbook of experience sampling methodology: A step‐by‐step guide to designing, conducting and analysing ESM studies. The center for Research on Experience sampling and Ambulatory methods Leuven (REAL).

[jcv270183-bib-0062] Nelson, K. L. , Davis, J. E. , & Corbett, C. F. (2022). Sleep quality: An evolutionary concept analysis. Nursing Forum, 57(1), 144–151. 10.1111/nuf.12659 34610163

[jcv270183-bib-0063] Nolen‐Hoeksema, S. (2012). Emotion regulation and psychopathology: The role of gender. Annual Review of Clinical Psychology, 8(1), 61–87. 10.1146/annurev-clinpsy-032511-143109 22035243

[jcv270183-bib-0064] Palmer, C. A. , & Alfano, C. A. (2017). Sleep and emotion regulation: An organizing, integrative review. Sleep Medicine Reviews, 31, 6–16. 10.1016/j.smrv.2015.12.006 26899742

[jcv270183-bib-0065] Parsons, C. E. , Schofield, B. , Batziou, S. E. , Ward, C. , & Young, K. S. (2022). Sleep quality is associated with emotion experience and adaptive regulation of positive emotion: An experience sampling study. Journal of Sleep Research, 31(4), 1–15. 10.1111/jsr.13533 35896512

[jcv270183-bib-0066] Paschke, L. M. , Dorfel, D. , Steimke, R. , Trempler, I. , Magrabi, A. , Ludwig, V. U. , Schubert, T. , Stelzel, C. , & Walter, H. (2016). Individual differences in self‐reported self‐control predict successful emotion regulation. Social Cognitive and Affective Neuroscience, 11(8), 1193–1204. 10.1093/scan/nsw036 PMC4967798 27013102

[jcv270183-bib-0067] Patel, P. K. , Leathem, L. D. , Currin, D. L. , & Karlsgodt, K. H. (2021). Adolescent neurodevelopment and vulnerability to psychosis. Biological Psychiatry, 89(2), 184–193. 10.1016/j.biopsych.2020.06.028 PMC9397132 32896384

[jcv270183-bib-0068] Paulus, F. W. , Ohmann, S. , Mohler, E. , Plener, P. , & Popow, C. (2021). Emotional dysregulation in children and adolescents with psychiatric disorders. A narrative review. Frontiers in Psychiatry, 12, 628252. 10.3389/fpsyt.2021.628252 PMC8573252 34759846

[jcv270183-bib-0069] Plessen, K. J. , Constanty, L. , Ranjbar, S. , Turri, F. , Miano, G. , Lepage, C. , & Urben, S. (2023). The role of self‐regulatory control processes in understanding aggressive ideations and behaviors: An experience sampling method study. Frontiers in Psychiatry, 13, 1058814. 10.3389/fpsyt.2022.1058814 PMC9893502 36741583

[jcv270183-bib-0070] Rouselle, P. , & Vigneau, F. (2016). Adaptation et validation d’une version brève en langue française du questionnaire d’impulsivité de Barratt (BIS‐15). European Review of Applied Psychology, 66(6), 317–324. 10.1016/j.erap.2016.05.001

[jcv270183-bib-0071] Saatchi, B. , Olshansky, E. F. , & Fortier, M. A. (2023). Irritability: A concept analysis. International Journal of Mental Health Nursing, 32(5), 1193–1210. 10.1111/inm.13140 36929104

[jcv270183-bib-0072] Sanchis‐Sanchis, A. , Grau, M. D. , Moliner, A. R. , & Morales‐Murillo, C. P. (2020). Effects of age and gender in emotion regulation of children and adolescents. Frontiers in Psychology, 11, 946. 10.3389/fpsyg.2020.00946 PMC7265134 32528367

[jcv270183-bib-0073] Selby, E. A. , Kranzler, A. , Panza, E. , & Fehling, K. B. (2016). Bidirectional‐compounding effects of rumination and negative emotion in predicting impulsive behavior: Implications for emotional cascades. Journal of Personality, 84(2), 139–153. 10.1111/jopy.12147 PMC6448804 25388298

[jcv270183-bib-0074] Shao, X. , Dong, Z. , Zhang, S. , Qiao, Y. , Zhang, H. , & Guo, H. (2025). The relationship between negative emotions and adjustment disorder in young adults: The mediating role of rumination and insomnia. Frontiers in Psychiatry, 16, 1474108. 10.3389/fpsyt.2025.1474108 PMC11891341 40066135

[jcv270183-bib-0075] Short, M. A. , & Louca, M. (2015). Sleep deprivation leads to mood deficits in healthy adolescents. Sleep Medicine, 16(8), 987–993. 10.1016/j.sleep.2015.03.007 26141007

[jcv270183-bib-0076] Silver, J. , Hawes, M. T. , Carlson, G. A. , & Klein, D. N. (2025). Irritability: Associations with real‐time affect dynamics, social interactions, and daily substance use in older adolescents. European Child & Adolescent Psychiatry, 34(2), 623–632. 10.1007/s00787-024-02504-9 38954054

[jcv270183-bib-0077] Snel, J. , & Lorist, M. M. (2011). Effects of caffeine on sleep and cognition. Progress in Brain Research, 190, 105–117. 10.1016/B978-0-444-53817-8.00006-2 21531247

[jcv270183-bib-0078] Solmi, M. , Fornaro, M. , Ostinelli, E. G. , Zangani, C. , Croatto, G. , Monaco, F. , Krinitski, D. , Fusar‐Poli, P. , & Correll, C. U. (2020). Safety of 80 antidepressants, antipsychotics, anti‐attention‐deficit/hyperactivity medications and mood stabilizers in children and adolescents with psychiatric disorders: A large scale systematic meta‐review of 78 adverse effects. World Psychiatry, 19(2), 214–232. 10.1002/wps.20765 PMC7215080 32394557

[jcv270183-bib-0079] Sukhodolsky, D. G. , Golub, A. , & Cromwell, E. N. (2001). Development and validation of the anger rumination scale. Personality and Individual Differences, 31(5), 689–700. 10.1016/S0191-8869(00)00171-9

[jcv270183-bib-0080] Takebe, M. , Takahashi, F. , & Sato, H. (2016). Anger rumination as a risk factor for trait anger and anger‐in: A longitudinal study. Personality and Individual Differences, 101, 451–455. 10.1016/j.paid.2016.06.038

[jcv270183-bib-0081] Tangney, J. P. , Baumeister, R. F. , & Boone, A. L. (2004). High self‐control predicts good adjustment, less pathology, better grades, and interpersonal success. Journal of Personality, 72(2), 271–322. 10.1111/j.0022-3506.2004.00263.x 15016066

[jcv270183-bib-0082] Tate, A. D. , Fertig, A. R. , de Brito, J. N. , Ellis, E. M. , Carr, C. P. , Trofholz, A. , & Berge, J. M. (2024). Momentary factors and study characteristics associated with participant burden and protocol adherence: Ecological momentary assessment. JMIR Formative Research, 8(1), e49512. 10.2196/49512 PMC11079761 38656787

[jcv270183-bib-0083] te Brinke, L. W. , Menting, A. T. A. , Schuiringa, H. D. , Zeman, J. , & Deković, M. (2020). The structure of emotion regulation strategies in adolescence: Differential links to internalizing and externalizing problems. Social Development, 30(2), 536–553. 10.1111/sode.12496

[jcv270183-bib-0084] Tetering, M. , Laan, A. M. V. , Kogel, C. H. , Groot, R. H. M. , & Jolles, J. (2020). Sex differences in self‐regulation in early, middle and late adolescence: A large‐scale cross‐sectional study. PLoS One, 15(1), e0227607. 10.1371/journal.pone.0227607 PMC6957194 31929576

[jcv270183-bib-0085] Thompson, R. A. (1994). Emotion regulation: A theme in search of definition. In The development of emotion regulation (Vol. 59, pp. 25–52). Monographs of the Society for Research in Child Development. 7984164

[jcv270183-bib-0086] Toohey, M. J. , & DiGiuseppe, R. (2017). Defining and measuring irritability: Construct clarification and differentiation. Clinical Psychology Review, 53, 93–108. 10.1016/j.cpr.2017.01.009 28284170

[jcv270183-bib-0087] Tseng, W. L. , Naim, R. , Chue, A. , Shaughnessy, S. , Meigs, J. , Pine, D. S. , Leibenluft, E. , Kircanski, K. , & Brotman, M. A. (2023). Network analysis of ecological momentary assessment identifies frustration as a central node in irritability. Journal of Child Psychology and Psychiatry, 64(8), 1212–1221. 10.1111/jcpp.13794 PMC10615387 36977629

[jcv270183-bib-0088] Turri, F. , Jones, A. , Constanty, L. , Ranjbar, S. , Drexl, K. , Miano, G. , Lepage, C. , Plessen, K. J. , & Urben, S. (2024). Self‐regulatory control processes in youths: A temporal network analysis approach. JCPP Advances, 4(1), e12200. 10.1002/jcv2.12200 PMC10933645 38486949

[jcv270183-bib-0089] Uccella, S. , Cordani, R. , Salfi, F. , Gorgoni, M. , Scarpelli, S. , Gemignani, A. , Geoffroy, P. A. , De Gennaro, L. , Palagini, L. , Ferrara, M. , & Nobili, L. (2023). Sleep deprivation and insomnia in adolescence: Implications for mental health. Brain Sciences, 13(4), 569. 10.3390/brainsci13040569 PMC10136689 37190534

[jcv270183-bib-0090] Vandekerckhove, M. , & Wang, Y. L. (2018). Emotion, emotion regulation and sleep: An intimate relationship. AIMS Neuroscience, 5(1), 1–17. 10.3934/Neuroscience.2018.1.1 PMC7181893 32341948

[jcv270183-bib-0091] Van Veen, M. M. , Lancel, M. , Beijer, E. , Remmelzwaal, S. , & Rutters, F. (2021). The association of sleep quality and aggression: A systematic review and meta‐analysis of observational studies. Sleep Medicine Reviews, 59, 101500. 10.1016/j.smrv.2021.101500 34058519

[jcv270183-bib-0092] Vermeersch, S. , & Fombonne, E. (1997). Le Child Behavior Checklist: Résultats préliminaires de la standardisation de la version française. Neuropsychiatrie de l'Enfance et de l'Adolescence, 45(10), 615–620.

[jcv270183-bib-0093] Vidal‐Ribas, P. , Brotman, M. A. , Valdivieso, I. , Leibenluft, E. , & Stringaris, A. (2016). The status of irritability in psychiatry: A conceptual and quantitative review. Journal of the American Academy of Child & Adolescent Psychiatry, 55(7), 556–570. 10.1016/j.jaac.2016.04.014 PMC4927461 27343883

[jcv270183-bib-0094] Vidal‐Ribas, P. , Krebs, G. , Silver, J. , Tseng, W. L. , Ford, T. , & Stringaris, A. (2025). The hidden burden: Self‐reported irritability in adolescent girls signals higher psychiatric risk. BMC Public Health, 25(1), 1832. 10.1186/s12889-025-23076-6 PMC12087224 40389896

[jcv270183-bib-0095] Vreugdenhil, C. , van den Brink, W. , Ferdinand, R. , Wouters, L. , & Doreleijers, T. (2006). The ability of YSR scales to predict DSM/DISC‐C psychiatric disorders among incarcerated male adolescents. European Child & Adolescent Psychiatry, 15(2), 88–96. 10.1007/s00787-006-0497-8 16463011

[jcv270183-bib-0096] Vroegindeweij, A. , Swart, J. F. , Houtveen, J. , Eijkelkamp, N. , Van De Putte, E. M. , Wulffraat, N. M. , & Nijhof, S. L. (2022). Identifying disrupted biological factors and patient‐tailored interventions for chronic fatigue in adolescents and young adults with Q‐fever fatigue syndrome, chronic fatigue syndrome and juvenile idiopathic arthritis (QFS‐study): Study protocol for a randomized controlled trial with single‐subject experimental case series design. Trials, 23(1), 683. 10.1186/s13063-022-06620-2 PMC9389501 35986408

[jcv270183-bib-0097] Wang, W. , Zhu, Y. , Yu, H. , Wu, C. , Li, T. , Ji, C. , Jiang, Y. , & Ding, D. (2024). The impact of sleep quality on emotion regulation difficulties in adolescents: A chained mediation model involving daytime dysfunction, social exclusion, and self‐control. BMC Public Health, 24(1), 1862. 10.1186/s12889-024-19400-1 PMC11241850 38992632

[jcv270183-bib-0098] Watkins, E. R. , & Roberts, H. (2020). Reflecting on rumination: Consequences, causes, mechanisms and treatment of rumination. Behaviour Research and Therapy, 127, 103573. 10.1016/j.brat.2020.103573 32087393

[jcv270183-bib-0099] Whiting, C. , Bellaert, N. , Deveney, C. , & Tseng, W. L. (2023). Associations between sleep quality and irritability: Testing the mediating role of emotion regulation. Personality and Individual Differences, 213, 1–5. 10.1016/j.paid.2023.112322 PMC10978035 38549685

[jcv270183-bib-0100] Wiese, B. S. , Chaillié, O. , Noppeney, R. , & Stertz, A. M. (2020). Daily experiences of commuting strain and self‐control at work and at home: A diary study with train travelers. Journal of Personnel Psychology, 19(3), 125–134. 10.1027/1866-5888/a000256

[jcv270183-bib-0101] Wrzus, C. , & Neubauer, A. B. (2023). Ecological momentary assessment: A meta‐analysis on designs, samples, and compliance across research fields. Assessment, 30(3), 825–846. 10.1177/10731911211067538 PMC9999286 35016567

[jcv270183-bib-0102] Ye, H. , Jiang, N. , He, S. , & Fan, F. (2024). Sleep disturbance and internalizing symptoms in adolescents: A moderated mediation model of self‐control and mindfulness. BMC Psychiatry, 24(1), 310. 10.1186/s12888-024-05750-y PMC11040859 38658904

[jcv270183-bib-0103] Yu, Q. , Hodgdon, E. A. , Kryza‐Lacombe, M. , Osuna, L. , Bozzetto, L. E. , Ciro, D. , Wakschlag, L. S. , & Wiggins, J. L. (2023). Roads diverged: Developmental trajectories of irritability from toddlerhood through adolescence. Journal of the American Academy of Child & Adolescent Psychiatry, 62(4), 457–471. 10.1016/j.jaac.2022.07.849 PMC10112490 36334890

[jcv270183-bib-0104] Zhang, Y. , MacNeill, L. A. , Edwards, R. C. , Burns, J. L. , Zola, A. R. , Poleon, R. B. , Nili, A. N. , Giase, G. M. , Ahrenholtz, R. M. , Wiggins, J. L. , Norton, E. S. , & Wakschlag, L. S. (2024). Developmental trajectories of irritability across the transition to toddlerhood: Associations with effortful control and psychopathology. Research on Child and Adolescent Psychopathology, 52(1), 125–139. 10.1007/s10802-023-01098-1 37410219

[jcv270183-bib-0105] Zik, J. , Deveney, C. M. , Ellingson, J. M. , Haller, S. P. , Kircanski, K. , Cardinale, E. M. , Brotman, M. A. , & Stoddard, J. (2022). Understanding irritability in relation to anger, aggression, and informant in a pediatric clinical population. Journal of the American Academy of Child & Adolescent Psychiatry, 61(5), 711–720. 10.1016/j.jaac.2021.08.012 PMC8863995 34438022

