# Supplementary material for: The moderating role of anger rumination and self‐control in the day‐to‐day associations between irritability and sleep in male adolescents: An exploratory experience sampling method study
Source: JCPP Adv. 2026 Oct 29:e70183. Online ahead of print. doi: 10.1002/jcv2.70183 (PMC13718871; doi:10.1002/jcv2.70183)
Supplement: Supplementary file 1 [file JCV2-9999-e70183-s001.docx]

**The moderating role of anger rumination and self-control in the day-to-day associations between irritability and sleep in male adolescents: An exploratory experience sampling method study**

**Supporting Information**

Table S1. Multilevel mixed-effects model predicting daily irritability, excluding baseline sleep difficulties as a covariate.

|  | **Daily irritability** | | | | |
| --- | --- | --- | --- | --- | --- |
| *Predictors* | *Estimates* | *Std. Beta* | *CI* | *Std. CI* | *p* |
| (Intercept) | 48.31 | 0.00 | 13.28 – 83.34 | 0.00 – 0.00 | **0.007** |
| Age | -1.65 | -0.31 | -2.97 – -0.32 | -0.56 – -0.06 | **0.015** |
| Trait maladaptive ER | 0.28 | 0.32 | 0.08 – 0.48 | 0.10 – 0.55 | **0.005** |
| Trait sleep quantity | -1.04 | -0.13 | -3.05 – 0.97 | -0.38 – 0.12 | 0.312 |
| Trait irritability | 2.99 | 0.21 | -0.71 – 6.69 | -0.05 – 0.46 | 0.113 |
| Trait self-control | 0.13 | 0.12 | -0.12 – 0.38 | -0.11 – 0.35 | 0.310 |
| Anger rumination (bp) | 0.09 | 0.17 | -0.05 – 0.23 | -0.08 – 0.41 | 0.191 |
| Self-control (bp) | -0.26 | -0.52 | -0.38 – -0.13 | -0.77 – -0.27 | **<0.001** |
| Sleep (bp) | -0.80 | -0.11 | -2.75 – 1.15 | -0.38 – 0.16 | 0.420 |
| Anger rumination (wp) | 0.26 | 0.25 | 0.17 – 0.35 | 0.17 – 0.34 | **<0.001** |
| Self-control (wp) | -0.24 | -0.18 | -0.35 – -0.12 | -0.27 – -0.10 | **<0.001** |
| Sleep (wp) | -9.37 | -0.92 | -13.36 – -5.38 | -1.31 – -0.53 | **<0.001** |
| Anger rumination (bp) × wp sleep | 0.11 | 0.18 | 0.04 – 0.18 | 0.07 – 0.30 | **0.002** |
| Self-control (bp) × Sleep (wp) | 0.10 | 0.75 | 0.05 – 0.15 | 0.38 – 1.12 | **<0.001** |
| **Random Effects** | | | | | |
| σ^2^ | 122.10 | | | | |
| τ_00_ _id_task_ | 21.18 | | | | |
| ICC | 0.15 | | | | |
| N _id_task_ | 56 | | | | |
| Observations | 465 | | | | |
| Marginal R^2^ / Conditional R^2^ | 0.320 / 0.420 | | | | |

Table S2. Multilevel mixed-effects model predicting daily sleep quality, excluding baseline sleep difficulties as a covariate.

|  | **Daily sleep quality** | | | | |
| --- | --- | --- | --- | --- | --- |
| *Predictors* | *Beta* | *Std. Beta* | *CI* | *Std. CI* | *p* |
| (Intercept) | 1.98 | 0.00 | -3.42 – 7.38 | 0.00 – 0.00 | 0.472 |
| Age | 0.01 | 0.01 | -0.20 – 0.21 | -0.29 – 0.30 | 0.951 |
| Trait maladaptive ER | -0.01 | -0.07 | -0.04 – 0.02 | -0.35 – 0.21 | 0.617 |
| Trait sleep quantity | 0.15 | 0.14 | -0.14 – 0.44 | -0.14 – 0.42 | 0.313 |
| Trait irritability | -0.40 | -0.22 | -0.94 – 0.14 | -0.51 – 0.08 | 0.147 |
| Trait self-control | 0.01 | 0.10 | -0.02 – 0.05 | -0.16 – 0.37 | 0.437 |
| Anger rumination (bp) | -0.01 | -0.10 | -0.03 – 0.01 | -0.39 – 0.18 | 0.484 |
| Self-control (bp) | 0.02 | 0.29 | -0.00 – 0.04 | -0.03 – 0.62 | 0.075 |
| Irritability (bp) | -0.02 | -0.18 | -0.06 – 0.02 | -0.54 – 0.19 | 0.345 |
| Lag anger rumination (wp) | -0.00 | -0.01 | -0.01 – 0.01 | -0.11 – 0.08 | 0.800 |
| Lag irritability (wp) | -0.01 | -0.11 | -0.02 – -0.00 | -0.20 – -0.01 | **0.033** |
| Lag self-control (wp) | 0.00 | 0.02 | -0.01 – 0.01 | -0.07 – 0.11 | 0.681 |
| **Random Effects** | | | | | |
| σ^2^ | 1.54 | | | | |
| τ_00_ _id_task_ | 0.58 | | | | |
| ICC | 0.27 | | | | |
| N _id_task_ | 56 | | | | |
| Observations | 462 | | | | |
| Marginal R^2^ / Conditional R^2^ | 0.207 / 0.423 | | | | |

Table S3. Multilevel mixed-effects model predicting daily irritability from sleep quality (Model 1), including cross-level interactions with anger rumination and self-control (random-intercept model; comparison with random-slope models reported separately).

|  | **Daily irritability** | | |
| --- | --- | --- | --- |
| *Predictors* | *Beta* | *CI* | *p* |
| (Intercept) | 45.91 | 10.33 – 81.49 | **0.013** |
| Age | -1.64 | -2.93 – -0.36 | **0.015** |
| Trait maladaptive ER | 0.29 | 0.10 – 0.48 | **0.004** |
| Trait sleep difficulties | 0.70 | -3.07 – 4.47 | 0.713 |
| Trait sleep quantity | -0.88 | -3.03 – 1.27 | 0.421 |
| Trait irritability | 2.95 | -0.55 – 6.45 | 0.102 |
| Trait self-control | 0.14 | -0.10 – 0.37 | 0.264 |
| Anger rumination (bp) | 0.09 | -0.04 – 0.22 | 0.169 |
| Self-control (bp) | -0.26 | -0.37 – -0.14 | **<0.001** |
| Sleep (bp) | -0.80 | -2.65 – 1.06 | 0.398 |
| Anger rumination (wp) | 0.26 | 0.17 – 0.35 | **<0.001** |
| Self-control (wp) | -0.26 | -0.37 – -0.15 | **<0.001** |
| Sleep (wp) | -9.13 | -13.16 – -5.10 | **<0.001** |
| Anger rumination (bp) × wp sleep | 0.12 | 0.05 – 0.18 | **0.001** |
| Self-control (bp) × Sleep (wp) | 0.10 | 0.05 – 0.15 | **<0.001** |
| **Random Effects** | | | |
| σ^2^ | 123.45 | | |
| τ_00_ _id_task_ | 12.94 | | |
| ICC | 0.09 | | |
| N _id_task_ | 56 | | |
| Observations | 462 | | |
| Marginal R^2^ / Conditional R^2^ | 0.334 / 0.397 | | |

Table S4 Multilevel mixed-effects model predicting daily irritability from sleep quality, including cross-level interactions with anger rumination and self-control, and allowing the within-person effect of sleep to vary across individuals (random-slope model).

|  | **Daily irritability** | | |
| --- | --- | --- | --- |
| *Predictors* | *Beta* | *CI* | *p* |
| (Intercept) | 45.91 | 10.33 – 81.49 | **0.013** |
| Age | -1.64 | -2.93 – -0.36 | **0.015** |
| Trait maladaptive ER | 0.29 | 0.10 – 0.48 | **0.004** |
| Trait sleep difficulties | 0.70 | -3.07 – 4.47 | 0.713 |
| Trait sleep quantity | -0.88 | -3.03 – 1.27 | 0.421 |
| Trait irritability | 2.95 | -0.55 – 6.45 | 0.102 |
| Trait self-control | 0.14 | -0.10 – 0.37 | 0.264 |
| Anger rumination (bp) | 0.09 | -0.04 – 0.22 | 0.169 |
| Self-control (bp) | -0.26 | -0.37 – -0.14 | **<0.001** |
| Sleep (bp) | -0.80 | -2.65 – 1.06 | 0.398 |
| Anger rumination (wp) | 0.26 | 0.17 – 0.35 | **<0.001** |
| Self-control (wp) | -0.26 | -0.37 – -0.15 | **<0.001** |
| Sleep (wp) | -9.13 | -13.16 – -5.10 | **<0.001** |
| Anger rumination (bp) × wp sleep | 0.12 | 0.05 – 0.18 | **0.001** |
| Self-control (bp) × Sleep (wp) | 0.10 | 0.05 – 0.15 | **<0.001** |
| Observations | 462 | | |

Table S5. Model comparison between the random-intercept and random-slope (AR1) models predicting daily irritability, based on Akaike Information Criterion (AIC) and likelihood ratio tests, indicating no improvement in model fit with the inclusion of a random slope.

|  | **Model** | **df** | **AIC** | **BIC** | **logLik** | **Test** | **L.Ratio** | **p-value** |
| --- | --- | --- | --- | --- | --- | --- | --- | --- |
| Model 1 random-intercept | 1 | 18 | 3599.372 | 3673.812 | -1781.686 |  |  |  |
| Model 1 random-slope | 2 | 19 | 3601.372 | 3679.948 | -1781.686 | 1 vs 2 | 5.265711e-07 | 0.9994 |

Table S6. Multilevel mixed-effects model predicting daily sleep quality from baseline variables and lagged within-person predictors of irritability, anger rumination, and self-control (random-intercept model).

|  | **Daily sleep quality** | | |
| --- | --- | --- | --- |
| *Predictors* | *Beta* | *CI* | *p* |
| (Intercept) | 2.65 | -2.67 – 7.97 | 0.336 |
| Age | -0.01 | -0.20 – 0.19 | 0.944 |
| Trait maladaptive ER | -0.01 | -0.04 – 0.02 | 0.628 |
| Trait sleep difficulties | -0.18 | -0.72 – 0.36 | 0.513 |
| Trait sleep quantity | 0.10 | -0.21 – 0.41 | 0.516 |
| Trait irritability | -0.38 | -0.88 – 0.12 | 0.142 |
| Trait self-control | 0.01 | -0.02 – 0.05 | 0.402 |
| Anger rumination (bp) | -0.01 | -0.03 – 0.01 | 0.451 |
| Self-control (bp) | 0.02 | -0.00 – 0.04 | 0.058 |
| Irritability (bp) | -0.02 | -0.06 – 0.02 | 0.331 |
| Lag anger rumination (wp) | -0.00 | -0.01 – 0.01 | 0.844 |
| Lag irritability (wp) | -0.01 | -0.02 – -0.00 | **0.033** |
| Lag self-control (wp) | 0.00 | -0.01 – 0.01 | 0.818 |
| **Random Effects** | | | |
| σ^2^ | 1.56 | | |
| τ_00_ _id_task_ | 0.42 | | |
| ICC | 0.21 | | |
| N _id_task_ | 56 | | |
| Observations | 462 | | |
| Marginal R^2^ / Conditional R^2^ | 0.219 / 0.385 | | |

Table S7. Multilevel mixed-effects model predicting daily sleep quality from baseline variables and lagged within-person predictors of irritability, anger rumination, and self-control, allowing the within-person effect of irritability to vary across individuals (random-slope model).

|  | **Daily sleep quality** | | |
| --- | --- | --- | --- |
| *Predictors* | *Beta* | *CI* | *p* |
| (Intercept) | 2.65 | -2.67 – 7.97 | 0.336 |
| Age | -0.01 | -0.20 – 0.19 | 0.944 |
| Trait maladaptive ER | -0.01 | -0.04 – 0.02 | 0.628 |
| Trait sleep difficulties | -0.18 | -0.72 – 0.36 | 0.513 |
| Trait sleep quantity | 0.10 | -0.21 – 0.41 | 0.516 |
| Trait irritability | -0.38 | -0.88 – 0.12 | 0.142 |
| Trait self-control | 0.01 | -0.02 – 0.05 | 0.402 |
| Anger rumination (bp) | -0.01 | -0.03 – 0.01 | 0.451 |
| Self-control (bp) | 0.02 | -0.00 – 0.04 | 0.058 |
| Irritability (bp) | -0.02 | -0.06 – 0.02 | 0.331 |
| Lag anger rumination (wp) | -0.00 | -0.01 – 0.01 | 0.844 |
| Lag irritability (wp) | -0.01 | -0.02 – -0.00 | **0.033** |
| Lag self-control (wp) | 0.00 | -0.01 – 0.01 | 0.818 |
| Observations | 462 | | |

Table S8. Comparison of random-intercept and random-slope models predicting daily sleep quality, based on AIC and likelihood ratio tests, indicating no improvement in model fit with the inclusion of a random slope.

|  | **Model** | **df** | **AIC** | **BIC** | **logLik** | **Test** | **L.Ratio** | **p-value** |
| --- | --- | --- | --- | --- | --- | --- | --- | --- |
| Model 2 random-intercept | 1 | 16 | 1606.396 | 1672.565 | -787.1978 |  |  |  |
| Model 2 random-slope | 2 | 17 | 1608.396 | 1678.700 | -787.1978 | 1 vs 2 | 2.129464e-07 | 0.9996 |

Table S9. Multilevel mixed-effects model predicting daily irritability, excluding age as a covariate.

|  | **Daily irritability** | | | | |
| --- | --- | --- | --- | --- | --- |
| *Predictors* | *Beta* | *Std. Beta* | *CI* | *Std. CI* | *p* |
| (Intercept) | 8.63 | 0.00 | -15.02 – 32.28 | 0.00 – 0.00 | 0.473 |
| Trait maladaptive ER | 0.24 | 0.27 | 0.03 – 0.45 | 0.04 – 0.51 | **0.024** |
| Trait sleep difficulties | 1.89 | 0.12 | -2.22 – 5.99 | -0.14 – 0.37 | 0.367 |
| Trait sleep quantity | 0.56 | 0.07 | -1.49 – 2.60 | -0.18 – 0.32 | 0.594 |
| Trait irritability | 3.16 | 0.22 | -0.75 – 7.07 | -0.05 – 0.49 | 0.113 |
| Trait self-control | 0.13 | 0.12 | -0.13 – 0.40 | -0.12 – 0.37 | 0.330 |
| Anger rumination (bp) | 0.11 | 0.19 | -0.04 – 0.25 | -0.07 – 0.45 | 0.159 |
| Self-control (bp) | -0.23 | -0.47 | -0.36 – -0.11 | -0.73 – -0.21 | **<0.001** |
| Sleep (bp) | -0.82 | -0.11 | -2.88 – 1.25 | -0.40 – 0.17 | 0.438 |
| Anger rumination (wp) | 0.26 | 0.25 | 0.17 – 0.35 | 0.17 – 0.34 | **<0.001** |
| Self-control (wp) | -0.23 | -0.18 | -0.34 – -0.12 | -0.26 – -0.09 | **<0.001** |
| Sleep (wp) | -9.37 | -0.92 | -13.36 – -5.38 | -1.31 – -0.53 | **<0.001** |
| Anger rumination (bp) × wp sleep | 0.11 | 0.18 | 0.04 – 0.18 | 0.07 – 0.30 | **0.002** |
| Self-control (bp) × Sleep (wp) | 0.10 | 0.75 | 0.05 – 0.15 | 0.38 – 1.12 | **<0.001** |
| **Random Effects** | | | | | |
| σ^2^ | 122.04 | | | | |
| τ_00_ _id_task_ | 25.22 | | | | |
| ICC | 0.17 | | | | |
| N _id_task_ | 56 | | | | |
| Observations | 465 | | | | |
| Marginal R^2^ / Conditional R^2^ | 0.303 / 0.422 | | | | |
